# Methane production and oxidation potentials along a fen‐bog gradient from southern boreal to subarctic peatlands in Finland

**DOI:** 10.1111/gcb.15740

**Published:** 2021-06-28

**Authors:** Hui Zhang, Eeva‐Stiina Tuittila, Aino Korrensalo, Anna M. Laine, Salli Uljas, Nina Welti, Johanna Kerttula, Marja Maljanen, David Elliott, Timo Vesala, Annalea Lohila

**Affiliations:** ^1^ Institute for Atmospheric and Earth System Research (INAR) Department of Physics University of Helsinki Helsinki Finland; ^2^ Helsinki Institute of Sustainability Science (HELSUS) Helsinki Finland; ^3^ Department of Forest Sciences University of Eastern Finland Joensuu Finland; ^4^ Department of Ecology and Genetics University of Oulu Oulu Finland; ^5^ Geological Survey of Finland Kuopio Finland; ^6^ Department of Environmental and Biological Sciences University of Eastern Finland Kuopio Finland; ^7^ Environmental Sustainability Research Centre University of Derby Derby UK; ^8^ Institute for Atmospheric and Earth System Research (INAR) Department of Forest Sciences University of Helsinki Helsinki Finland; ^9^ Yugra State University Khanty‐Mansiysk Russia; ^10^ Climate System Research Finnish Meteorological Institute Helsinki Finland; ^11^ Present address: Commonwealth Scientific and Industrial Research Organization Urrbrae SA Australia

**Keywords:** bogs, fens, global warming, methane, peat property, production and oxidation, temperature response, vegetation

## Abstract

Methane (CH_4_) emissions from northern peatlands are projected to increase due to climate change, primarily because of projected increases in soil temperature. Yet, the rates and temperature responses of the two CH_4_ emission‐related microbial processes (CH_4_ production by methanogens and oxidation by methanotrophs) are poorly known. Further, peatland sites within a fen‐bog gradient are known to differ in the variables that regulate these two mechanisms, yet the interaction between peatland type and temperature lacks quantitative understanding. Here, we investigated potential CH_4_ production and oxidation rates for 14 peatlands in Finland located between c. 60 and 70°N latitude, representing bogs, poor fens, and rich fens. Potentials were measured at three different temperatures (5, 17.5, and 30℃) using the laboratory incubation method. We linked CH_4_ production and oxidation patterns to their methanogen and methanotroph abundance, peat properties, and plant functional types. We found that the rich fen‐bog gradient‐related nutrient availability and methanogen abundance increased the temperature response of CH_4_ production, with rich fens exhibiting the greatest production potentials. Oxidation potential showed a steeper temperature response than production, which was explained by aerenchymous plant cover, peat water holding capacity, peat nitrogen, and sulfate content. The steeper temperature response of oxidation suggests that, at higher temperatures, CH_4_ oxidation might balance increased CH_4_ production. Predicting net CH_4_ fluxes as an outcome of the two mechanisms is complicated due to their different controls and temperature responses. The lack of correlation between field CH_4_ fluxes and production/oxidation potentials, and the positive correlation with aerenchymous plants points toward the essential role of CH_4_ transport for emissions. The scenario of drying peatlands under climate change, which is likely to promote *Sphagnum* establishment over brown mosses in many places, will potentially reduce the predicted warming‐related increase in CH_4_ emissions by shifting rich fens to *Sphagnum*‐dominated systems.

## Graphical abstract

We investigated potential CH_4_ production and oxidation rates at three temperature levels for 14 peatlands in Finland, representing bogs, poor fens and rich fens, and linked the potential patterns to their methanogen and methanotroph abundance, peat properties, and plant functional types. The bog‐rich fen gradient related nutrient availability and methanogen abundance increased the temperature response of CH_4_ production, with rich fens exhibiting the greatest production potentials. Oxidation potential showed a steeper temperature response than production, which was explained by aerenchymous plant cover, peat water holding capacity, peat nitrogen and sulphate content.

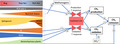

## 1 INTRODUCTION

Global estimates have revealed a change in the global methane (CH_4_) budget in the past decades, specifically, an increase in atmospheric CH_4_ concentrations until the early 2000s, a stabilization period until 2006, and an ongoing new rise afterwards (Dlugokencky et al., 2011; Nisbet et al., 2019; Rigby et al., 2008). This recent imbalance is considered to result from an increase in both fossil fuel production and biogenic emissions, although considerable uncertainties still exist in the global CH_4_ budget and its components (Bousquet et al., 2011; Ciais et al., 2013; Kirschke et al., 2013). Historical evidence shows that atmospheric CH_4_ concentration fluctuations have closely followed past climatic cycles (Blunier et al., 1995; Brook et al., 2000; Chappellaz et al., 1993), which suggest that biogenic emissions respond readily to climate changes and also have a crucial role in regulating climate. When the 28‐ to 34‐fold greater warming potential of CH_4_ compared to carbon dioxide (CO_2_) over a 100‐year horizon (IPCC, 2013) is considered, it is critical that the response of CH_4_ emissions from ecosystems to warming is fully evaluated, which will in turn help better predict future climates.

Northern peat‐accumulating wetlands, that is, peatlands, which cover approximately 15% of the boreal and arctic landscapes, represent the largest peatland area in the world, releasing 20–45 Tg CH_4_ into the atmosphere annually (Fletcher et al., 2004; Gorham, 1991). In the long term, they have acted as important CH_4_ sources and have impacted atmospheric CH_4_ concentrations since their initiation in the early Holocene, and during their extensive lateral expansion throughout the mid‐Holocene (Frolking & Roulet, 2007; Korhola et al., 2010; MacDonald et al., 2006). Modelling projections have suggested that global wetland CH_4_ emissions will increase throughout the 21st century and will have a positive feedback on global warming (Zhang et al., 2017). However, considerable uncertainties remain, largely due to the lack of data on the separate CH_4_ production and consumption/oxidation processes at an ecosystem‐scale. These are needed because such processes ultimately determine the magnitude of CH_4_ emissions to the atmosphere (Zhang et al., 2017). In addition, several studies have already shown that the activity and community composition of both CH_4_ producing (methanogens) and CH_4_ oxidizing (methanotrophs) microbes in peatlands are largely influenced by climate change‐related environmental conditions, such as water level and temperature (Larmola et al., 2010; Turetsky et al., 2008; Yrjälä et al., 2011). Despite this knowledge, the direction and magnitude of the responses of CH_4_ production and oxidation processes to such variables remain poorly understood and can differ greatly, which makes predicting the dynamics of the net CH_4_ flux in peatlands particularly challenging.

**FIGURE 1:**
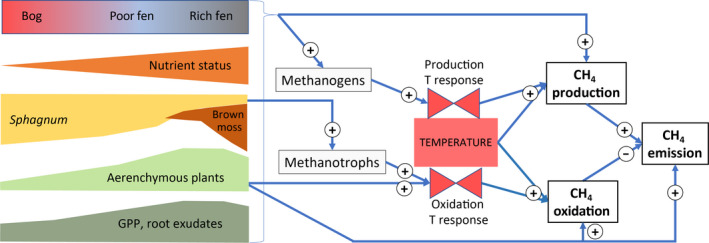
Hypothesized factors affecting peatland methane (CH_4_) production, oxidation, and emissions based on the literature. On the left‐hand side, the gradient from bogs to rich fens (red‐to‐blue rectangle) is associated with a shift in nutrient status and gradual shifts in peatland vegetation, which have an effect on gross primary productivity (GPP) and the amount and quality of litter and root exudates. The vertical width of the colored polygons (below the fen‐bog rectangle) represents the magnitude of these regulating factors along the peatland site type gradient. These differences from bogs to rich fens affect the composition and abundance of methanogens and methanotrophs. Although both the CH_4_ production and oxidation processes are temperature‐dependent, the steepness of their temperature response (red valves) is also modified by environmental factors. Aerenchymous vegetation cover has the capacity to increase CH_4_ oxidation by transporting oxygen into the anoxic zone below the water level but also directly increases CH_4_ emissions as this plant‐mediated transport can bypass the oxidation zone. *Sphagnum* mosses enhance CH_4_ oxidation by providing a habitat for methanotrophs. Water level was normalized in the sampling and, therefore, is not included in the illustration

Major controls on peatland CH_4_ emission process include water‐table level (approximately delineating the oxic‐anoxic boundary), availability and quality of organic substrates, and temperature (Walter & Heimann, 2000). In addition, the in situ vegetation composition strongly influences CH_4_ flux dynamics, both by adding labile carbon substrates for CH_4_ production (Ström et al., 2003) and by maintaining internal gas conduits that affect the production, oxidation, and transportation of CH_4_ from the peat to the atmosphere (Joabsson et al., 1999; Noyce et al., 2014). Furthermore, oxygen availability, for example, carried by flowing water or transported by aerenchymous plant species, can typically decrease CH_4_ emissions (Fritz et al., 2011; Zhang, Tuittila, et al., 2020; Figure 1), although, as mentioned above, aerenchymous plant species can also facilitate the transportation of CH_4_.

Methane fluxes in peatlands are widely divergent (Knox et al., 2019). Temporal variations (diurnal, seasonal, inter‐annual) in CH_4_ emissions, and equally large spatial variation between sites and within sites, at fixed measurement points have all been reported (Turetsky et al., 2014). In general, peatland types have been shown to clearly differ in the magnitude of CH_4_ emissions (Knox et al., 2019; Moore & Knowles, 1990; Treat et al., 2018), with fens displaying greater emissions than bogs due to enhanced methanogenic activity (Juottonen et al., 2005) and a greater litter degradation rate (Aerts et al., 1999). It has been suggested that the various peatland types within the boreal zone may show differing microbial activity and community structure; for example, rich fens contain more methanogens and *Sphagnum*‐dominated peatlands contain more methanotrophs, which could result in varying response of CH_4_ emissions to global change factors (Jaatinen et al., 2005, 2007; Figure 1).

In peatlands, the response of CH_4_ emissions to temperature appears to be somewhat unpredictable. Most studies report a clear dependence of CH_4_ emissions on the soil temperature (e.g., Christensen et al., 2003; Mastepanov et al., 2013; Treat et al., 2007). Likewise, biogeochemical models consider soil temperature as the main driver of CH_4_ emissions (e.g., Bridgham et al., 2013; Walter & Heimann, 2000). However, most of the derived associations between temperature and CH_4_ emissions were based on seasonal field datasets that are unable to separate the role of the two mechanistic processes of CH_4_ emissions, that is, CH_4_ production and oxidation. In addition, different temperature responses of CH_4_ fluxes throughout the growing season have also been observed (Pypker et al., 2013). Furthermore, experimental ecosystem warming studies have shown contradictory responses to warming from large increases in CH_4_ emissions (Hopple et al., 2020) to no change (Laine et al., 2019; Peltoniemi et al., 2016). Recent evidence indicates that some northern peatlands are becoming drier due to increased rates of evapotranspiration (Helbig et al., 2020; Swindles et al., 2019; Zhang, Väliranta, et al., 2020), while wetter conditions may also occur due to increased summer rainfall (Charman et al., 2007) and permafrost thaw (e.g., Sim et al., 2021; Zhang et al., 2018), for example. The impacts of warming, under different moisture regimes, on CH_4_ emissions might not be straightforward, even though drying alone could reduce both CH_4_ production and oxidation rates (Peltoniemi et al., 2016). Nevertheless, understanding how the environment modulates the temperature response of CH_4_ production and oxidation will benefit the study of the interaction between temperature and moisture and is, therefore, crucial for further robust predictions of the global CH_4_ budget.

In this study, we aim to quantify how peat properties and plant functional types affect peatland CH_4_ production and oxidation, and influence their temperature responses using incubation experiments. We collected peat samples from 14 peatlands in Finland within approximately 10 latitudinal degrees, which represent three major peatland types within the boreal to subarctic zones: bogs, poor fens, and rich fens. Based on previously documented knowledge, we constructed a conceptual model (Figure 1) to visualize the expected patterns in our dataset. Specifically, we hypothesized that (1) fens have greater CH_4_ production potential rates than bogs due to the potentially greater abundance of methanogens (Juottonen et al., 2005), and (2) bogs and poor fens with *Sphagnum* carpets have greater oxidation potential rates due to greater abundance and activity of methanotrophs than rich fens where brown mosses dominate over *Sphagnum* (e.g., Putkinen et al., 2018). We tested these hypotheses by linking the CH_4_ production and oxidation potential rates and their temperature response to measured peat properties and plant functional type data. In addition, we expected greater CH_4_ fluxes in fens than in bogs at our sites due to the enhanced abundance of graminoid plants following the pattern reported in Turetsky et al. (2014). We also explored whether CH_4_ emission patterns in the same study sites (reported in the literature or unpublished; Table S1) could be connected to their CH_4_ production and oxidation potential rates. Our particular questions were: (1) What are the roles of peat properties and plant functional types in driving CH_4_ production and oxidation? (2) How does the temperature response vary for CH_4_ production and oxidation, and for different peatland types?

## 2 MATERIALS AND METHODS

### 2.1 Study sites

**FIGURE 2:**
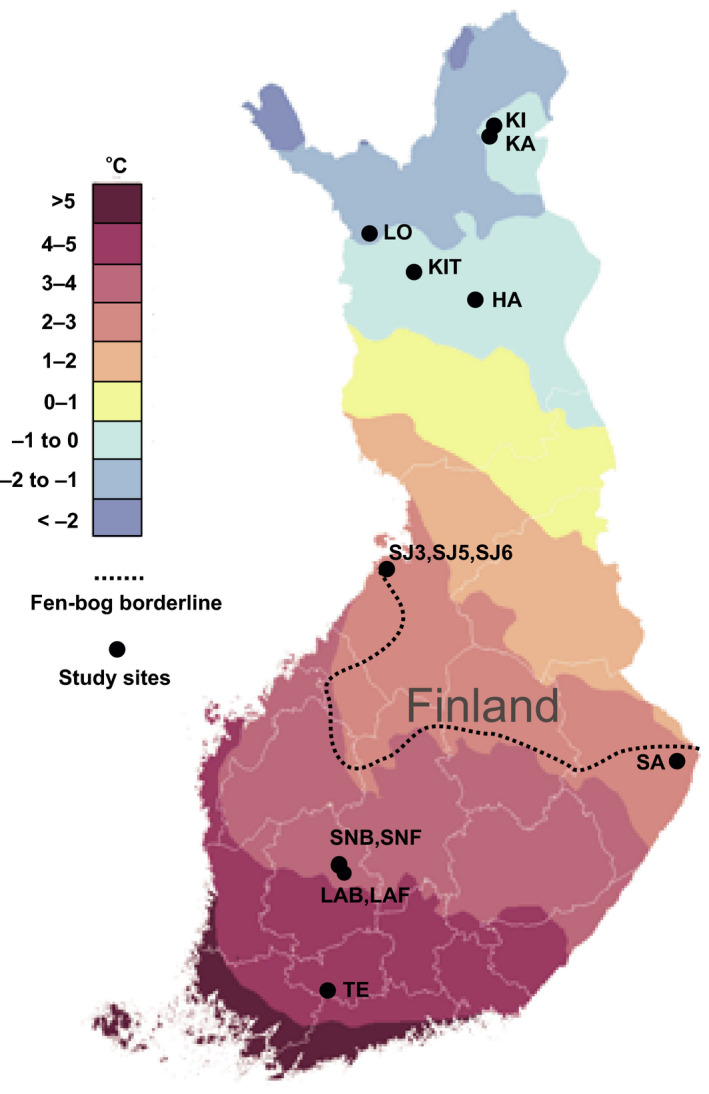
Location of the study sites. The base map was downloaded from https://en.ilmatieteenlaitos.fi/normal‐period and indicates the average annual temperature (1981–2010) gradient in Finland. Geographical division of the northern aapamire (fen) and southern raised bog zones (Väliranta et al., 2017) is outlined using a dashed line

In total, 14 study sites throughout Finland were selected (Table 1; Figure 2), where CH_4_ flux data (Table S1) already existed. The sites were located between 60 and 70°N, with a long‐term annual temperature range of 6℃ (range from −1.4 to 4.6℃; Table 1). These study sites were disaggregated based on their vegetation composition and relative position in the landscape into three peatland types with differing nutrient regimes, that is, ombrotrophic bog (fed solely by precipitation; nutrient poor; hereafter referred to as bog), oligotrophic fen (additionally fed by groundwater inputs; moderate nutrient regime; hereafter referred to as poor fen), and meso/meso‐eutrophic/eutrophic fen (fed by groundwater; nutrient‐rich; hereafter referred to as rich fen; Table 1).

**TABLE 1:** Study site information

Peatland type	Site	Site code	Latitude (°N)	Longitude (°E)	Annual T (°C)	Annual P (mm)
Bog	Tervalamminsuo	TE	60.65	23.97	4.6	627
	Lakkasuo bog	LAB	61.79	24.31	4.2	711
	Siikaneva bog	SNB	61.84	24.17	4.2	711
	Salmisuo	SA	62.77	30.97	3.0	613
	Siikajoki 6	SJ6	64.72	24.70	2.6	539
Poor fen	Siikaneva fen	SNF	61.83	24.20	4.2	711
	Siikajoki 5	SJ5	64.74	24.72	2.6	539
Rich fen	Lakkasuo fen	LAF	61.80	24.32	4.2	711
	Siikajoki 3	SJ3	64.76	24.68	2.6	539
	Halssiaapa	HA	67.37	26.65	−0.4	527
	Kittilä	KIT	67.70	25.12	−0.6	506
	Lompolojänkkä	LO	67.98	24.35	−1.4	484
	Kaamanen	KA	69.14	27.27	−0.4	472
	Kiposuo	KI	69.30	27.53	−1.0	395

Note

Annual temperature (T) and precipitation (P) data are average values for the period 1981–2010 from the nearest meteorological stations (Pirinen et al., 2012).

Our sampling of bogs and fens reflects the distribution of these peatland types in Finland. Geographically, Finland can be roughly divided into raised bogs in the south and northern aapamire (fen) zones (Figure 2). Raised bogs can also be found in the aapamire zone but they are rare, and none have been used for CH_4_ flux measurements in northern Finland. Consequently, all our bog sites are located in southern Finland. Within the raised bog zone, fens are commonly located at the margin of the peatland massif, while the center is characterized by bog vegetation. As CH_4_ flux measurements have been carried out in the southern fens, we were able to sample both fens and bogs in the south.

### 2.2 Field sampling

From the end of July 2015 to the end of August 2015, triplicate peat samples were collected from 0 to 10 cm below the surface of the living moss layer at each site. We sampled lawns at each of the sites that had a water level at 0–5 cm below the moss surface to remove the variation related to the water table. The peat cores were homogenized and then separated into triplicate peat samples under anoxic conditions using an anoxic glove box to preserve the initial anoxic conditions (Kettunen et al., 1999). The samples were kept cooled (at 4℃) and anoxic during transportation and in the laboratory before analysis or incubation. At each site, we measured water‐table depth and estimated the projection cover for each plant species (Table S3) within a 30‐cm circular frame at the three sampling points. Plant functional type cover, that is, sedge, herb, shrub, *Sphagnum* moss, brown moss, and the cover of aerenchymous plants, were also recorded at each sampling point.

### 2.3 Laboratory incubation

CH_4_ production and oxidation potentials were quantified at three temperatures (5, 17.5, and 30℃) for each sample using oxic and anoxic incubation laboratory experiments. The chosen temperature levels were based on actual field temperatures in Finland, where the minimum temperature during summer is approximately 5℃ (1981–2010; https://en.ilmatieteenlaitos.fi/normal‐period) and the maximum temperature can exceed 30℃, as evidenced in the extremely warm year 2010 (37.2℃ on 29 July, Liperi, Finland). This 5–30℃ range is also the common summer temperature range across the boreal zone where most of the global peatlands are distributed. Two treatments (under anoxic and oxic conditions) were used to calculate the respective CH_4_ production and oxidation potentials of the peat. The experiments were kept short (10 days) in order to undertake the measurements before the microbial community could thermally adapt to the various incubation temperatures, even though under real field conditions thermal adaptation is likely to occur given the relatively slow process of any climate changes.

The anoxic treatment was prepared using 100‐ml glass bottles, each containing 7–10 g peat (fresh weight; FW), which were closed under anoxic conditions, adjusted with 50‐ml overpressure using N_2_, and stored at the respective temperatures. The oxic treatment was prepared using 500‐ml glass bottles, each containing 15–20 g_FW_ peat, which were filled and closed under anoxic conditions, but were re‐opened in the laboratory to establish oxic conditions. All bottles were then re‐closed and adjusted to c. 5000 µl L^−1^ (5000 ppm) initial CH_4_ concentration and 120‐ml overpressure using N_2_. This concentration was chosen to be comparable to the in‐situ measurements at 10–15‐cm depth in peat (unpublished data from Siikaneva and Lompolojänkkä). The oxygen concentration in the bottles was not regulated for the duration of the incubation. The bottles were sealed with rubber septa (to allow sampling with needles) and were secured with aluminum screw caps due to over pressure in the bottles.

The CH_4_ concentrations in the gas phase of all bottles were measured over the incubation process with sampling intervals between c. 24 and 70 h (Figure S1), determined with a gas chromatograph (Agilent 6890N, Agilent Technologies) equipped with an auto‐sampler (Gilson) and flame ionization (FID) detector. The CH_4_ production and oxidation rates were calculated from the linear fits of the headspace gas concentrations relative to time (i.e., relative time span from the first gas measurement) and normalized to dry weight (dw) of the peat. For each sample, seven to eight gas measurements were performed. If there were any indications of leaks, lags, or failures in the gas sampling or gas analysis, the results were excluded from the analyses. A more detailed description of the observed increasing/decreasing pattern of CH_4_ concentrations, and the calculation of production and oxidation potential rates can be found in Figure S1.

### 2.4 Peat property analysis

Soil nitrate (NO_3_^−^), nitrite (NO_2_^−^), ammonium (NH_4_^+^), chloride (Cl^−^), sulfate (SO_4_^2−^), iron (Fe^3+^), and phosphate (PO_4_^3−^) concentrations were measured from the soil extractions. For the analysis of anions, 15 ml peat and 50 ml H_2_O were shaken at 175 rpm for 1 h, filtered, and analyzed with an ion chromatograph (DX 120, Dionex Co.). Ammonium was analyzed by first extracting 15 ml peat with 50 ml 1 M KCl and analyzed using a spectrophotometer from the filtered KCl extracts according to Fawcett and Scott (1960). Gravimetric soil moisture was determined by drying the soil for 24 h at 105℃. Total carbon (C) and nitrogen (N) contents of the peat and stable isotope abundances (δ^13^C and δ^15^N) were determined with an elemental analyzer (Thermo Finnigan Flash EA 1112 Series). Bulk density (BD), water holding capacity (WHC), and loss on ignition (LOI) were determined from the samples. BD was defined as the dry weight of a known volume of peat. WHC was measured by measuring the dry weight of a peat sample, saturating it with water for 24 h and then weighing the sample again after allowing the excess water to drain. LOI was measured by burning a known dry weight of peat in an oven at 550℃ for 2 h and then reweighing the sample. All measurements were performed on pre‐incubation samples.

### 2.5 Microbial community analysis

Microbial community composition was determined by sequencing the microbial DNA in the peat samples. In contrast to the CH_4_ incubation and peat property analyses that were performed for all three replicated samples from each site, DNA analysis was conducted only on one sample per site. DNA for each sample was extracted from 250‐mg peat using a Mobio Powersoil kit (MO BIO Laboratories), and was then subjected to amplicon sequencing using the MiSeq platform, targeting the prokaryotic 16S rRNA gene and the fungal ITS. Sequence data were processed using the MR DNA analysis pipeline (MR DNA). Sequences were joined, depleted of barcodes, then sequences <150 bp and sequences with ambiguous base calls were removed. Sequences were denoised, operational taxonomic units (OTUs) were generated and chimeras were removed. OTUs were defined by clustering at 3% divergence (97% similarity). Final OTUs were taxonomically classified using BLASTn against a curated database derived from GreenGenes, RDPII and NCBI. The relative abundance of CH_4_‐production‐related Archaeal taxa (methanogens) and oxidation‐related bacterial taxa (methanotrophs; e.g., Nazaries et al., 2013) were quantified for each site as percentages of the sum of methanogens and methanotrophs. The calculation was performed on the taxonomically classified OTU data (number of reads). The proportions of methanogens and methanotrophs of the total prokaryotic microbiome were also quantified.

### 2.6 Data analysis

To assess the main variation in vegetation and its correlation with peat properties and plant functional types, we applied detrended correspondence analysis (DCA) on plant composition data and included plant functional types and peat properties (listed in Table S2) as supplementary variables. We selected DCA because it is a suitable method for capturing species turnover (i.e., elimination and replacement) along an extended environmental gradient, as in our dataset. To explore the patterns in methanogen and methanotroph abundance, we used principal component analysis (PCA) to visualize the microbial data.

To investigate the overall pattern of peat properties, plant functional types, CH_4_ production, and oxidation potentials, as well as CH_4_ fluxes measured in the field, we applied PCA to the peat properties and plant functional types with the supplementary variables of CH_4_ production and oxidation potentials at different temperatures, field fluxes, and peatland site types. The mean value of CH_4_ fluxes during the peak growing season was calculated for each site and used for the analysis. All ordination analyses were carried out using Canoco 5 (ter Braak & Šmilauer, 2012).

To test the differences between the peatland types in regard to peat properties, plant functional types, microbial abundance, and CH_4_ production and oxidation potentials at different temperatures, we applied mixed effect models for each variable separately, with the three peatland types as fixed predictors. We also tested the impact of peatland type on the temperature response of CH_4_ production and oxidation by constructing mixed effect models with peatland type, laboratory temperature, and their interaction as fixed predictors. CH_4_ data were log‐transformed before the analyses. Site and sample were included as nested random effects for peat properties, plant functional types, CH_4_ production and oxidation potentials, while only site was included as a random effect for CH_4_ fluxes and microbial data without replicates within site.

To quantify the impact of peat properties and plant functional types on CH_4_ production and oxidation potentials, as well as on the temperature response of these processes, we constructed two mixed effects models for production and oxidation potentials, respectively. CH_4_ data were log‐transformed before the analyses. First, we added incubation temperature as a fixed predictor to the model and defined which temperature response type (linear or polynomial) fitted the data better, based on *p*‐values of the fixed predictors in marginal ANOVA tests, as well as the residual distribution and AIC‐values of the alternative models. Next, we added fixed predictors into the models, one by one, based on our hypothesis of how variables regulate production and oxidation potentials (Figure 1). Both CH_4_ production and oxidation were hypothesized to change along the bog‐rich fen gradient that was represented by axis 1 in the PCA of environmental variables, gathering together the variation in several intercorrelated variables. Thus, we added PCA axis 1 sample scores as a fixed predictor in the models (Çamdevýren et al., 2005). Next, we added further potential predictors that were expected to improve the model, in addition to their contribution to PCA 1 axis, on the basis of our hypothetical illustration (Figure 1) and PCA of peat properties, plant functional types, and microbial communities (Figure 5). After each fixed predictor addition, we used the marginal ANOVA to test whether the model with a new predictor was significantly better than the simpler model. For each new fixed predictor, we also tested the significance of interaction with incubation temperature. In these mixed effects models, site and sample were included as nested random effects. The residuals of the final models were normally distributed around a mean of zero. All mixed effect models were developed using the lme() function in “nlme” package in R.3.6.1 (R Core Team, 2019).

## 3 RESULTS

### 3.1 Peat properties and plant functional types of different peatland types

**FIGURE 3:**
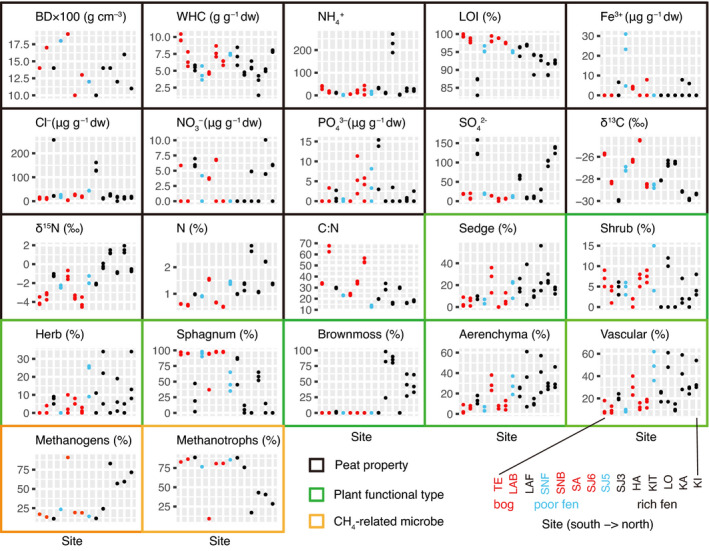
Measured peat properties, plant functional types, and methane (CH_4_)‐related microbes at each peatland site. Peat properties (with the exception of bulk density (BD)) and plant functional types for each site are composed of three replicates, but only one measurement per site for microbial community distribution. The sites (shown in the bottom‐right panel) are arranged in order from south to north, and peatland types are indicated by different colors, that is, bog (red), poor fens (blue), and rich fens (black)

As expected, our results indicated that vegetation composition, peat properties and plant functional types differed between the bogs and the fens (Figure 3; Figure S2; Table S2), which suggests that our sampling sites represent the typical conditions found in those peatland types. There were significant differences in several environmental factors between the three peatland types (Table S2). For example, bogs differed from rich fens in terms of greater water holding capacity (WHC), lower SO_4_^2−^, N content, and δ^15^N values, and reduced sedge and aerenchymous plant abundance, but resembled poor fens. Bogs and poor fens also displayed clearly greater *Sphagnum* cover than rich fens, while brown mosses were found only in the rich fens. C:N ratios were significantly higher in the bogs than in the fens, while the LOI values differed between the three types, being greatest in the bogs and lowest in the rich fens.

### 3.2 CH_4_‐related microbial community distribution

In the studied peatlands, CH_4_‐related microbes collectively accounted for 0.8%–6.8% of the total prokaryotic microbiome (Figure S3). Specifically, a much larger amount (6.8%) was detected for site SNB, while values ranging from 0.8% to 3.3% were observed for the other sites. The proportion of methanogens ranged from 0.1% to 1.9% across sites, with the exception of site SNB that had a much higher value (6.2%). The proportion of methanotrophs ranged from 0.4% to 1.6%.

Based on the taxonomic classification of OTUs, methanogens from 11 families in the Archaea group, and methanotrophs from two families in the bacteria group were detected (Figure S3). Within the CH_4_‐related taxa (i.e., sum of methanogens and methanotrophs), the most abundant methanogenic taxa were from families Methanosaetaceae (max. 65%), Methanoregulaceae (max. 18%), Methanobacteriaceae (max. 15%), and Methanospirillaceae (max. 10%). In addition, Methanospirillaceae was commonly recorded (c. 71%) at only one site (SNB), but abundances were <10% at the other sites. Methanotrophs were from families Methylocystaceae (max. 80%) and Methylococcaceae (max. 20%), with the former more commonly recorded. The four most northern rich fen sites (KIT, LO, KA and KI) and one bog site (SNB) contained more methanogens (>60%) but less methanotrophs (<40%) than the other sites (Figure S3).

**FIGURE 4:**
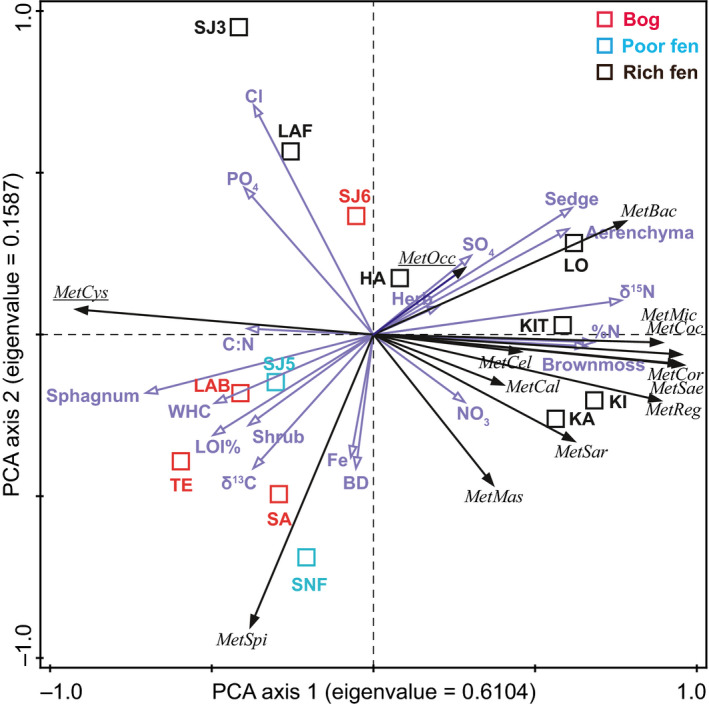
Principal component analysis (PCA) based on the relative abundance of methanogens and methanotrophs at each site (in black italic), with environmental data included as supplementary variables (in purple). The first two PCA axes explained 77% of the total variance in community structure. Methanogens and methanotrophs (underlined) are shown in black. The SNB site appeared as an outlier in the microbial dataset (Figure 6d) and was excluded from the analysis. Peatland types are indicated using red (bog), light blue (poor fen), and black (rich fen) site names. The full names of the analyzed variables can be found in Table S3 and the site codes are described in Table 1

The main variation in microbial community structure was related to the fen‐bog gradient (Figure 4). The abundance of all methanogens, with the exception of Methanospirillaceae, increased along the first axis from bog to rich fens (Figure 4). Methanospirillaceae was linked to the second axis, which separated the coastal SJ3 site from the others and was linearly correlated to Cl^−^ concentration (*p* < 0.001). The two methanotroph families were distributed at opposing ends of the fen‐bog gradient on axis 1. In contrast to Methylococcaceae, Methylocystaceae showed a distinct predominance in *Sphagnum*‐dominated habitats (*p* < 0.001).

### 3.3 CH_4_ production and oxidation potentials in relation to peat and vegetation properties and microbial communities

**FIGURE 5:**
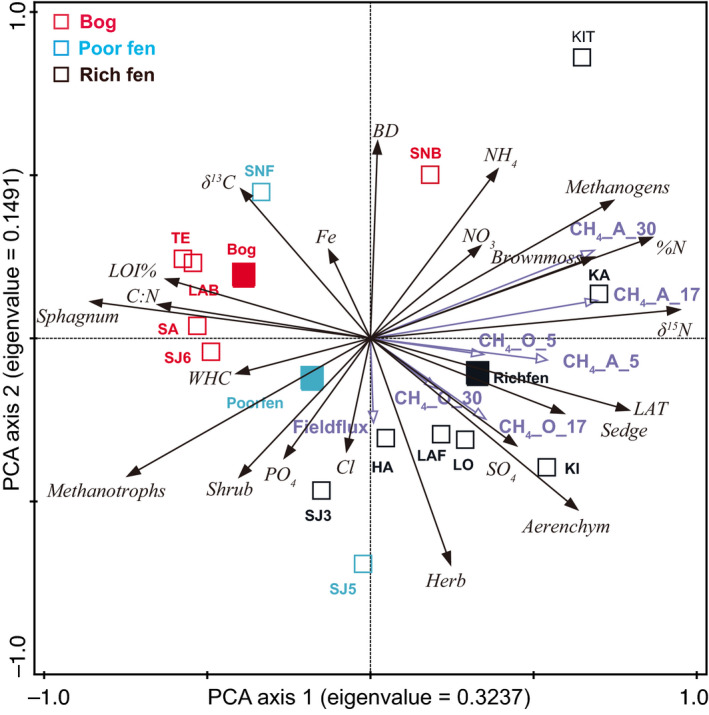
Principal component analysis (PCA) based on peat properties, plant functional types, and methane (CH_4_)‐related microbes (variables in black italic) with CH_4_ production and oxidation potentials at three different temperature levels and field fluxes included as supplementary variables (in purple). The first two axes explained 47.28% of the total variance. Peatland types are indicated using red (bog), light blue (poor fen) and black (rich fen) site names. The full names of the analyzed variables can be found in Table S3 and site codes are described in Table 1

The results from the PCA indicate that the observed variations in CH_4_ production (CH_4__A_temp.) and oxidation (CH_4__O_temp.) rates can be linked to peat properties and plant functional type conditions (Figure 5). The main variation in the data (axis 1 in Figure 5) from bogs to rich fens through poor fens was associated with increased CH_4_ production rates. The first axis was correlated with nutrient level, particularly N (e.g., N% and δ^15^N). CH_4_ oxidation rates were associated with both axes, with the second axis related to the variations in bulk density (BD) and PO_4_^3−^, for example, within the peatland type.

### 3.4 CH_4_ production and oxidation potentials of different peatland types

**FIGURE 6:**
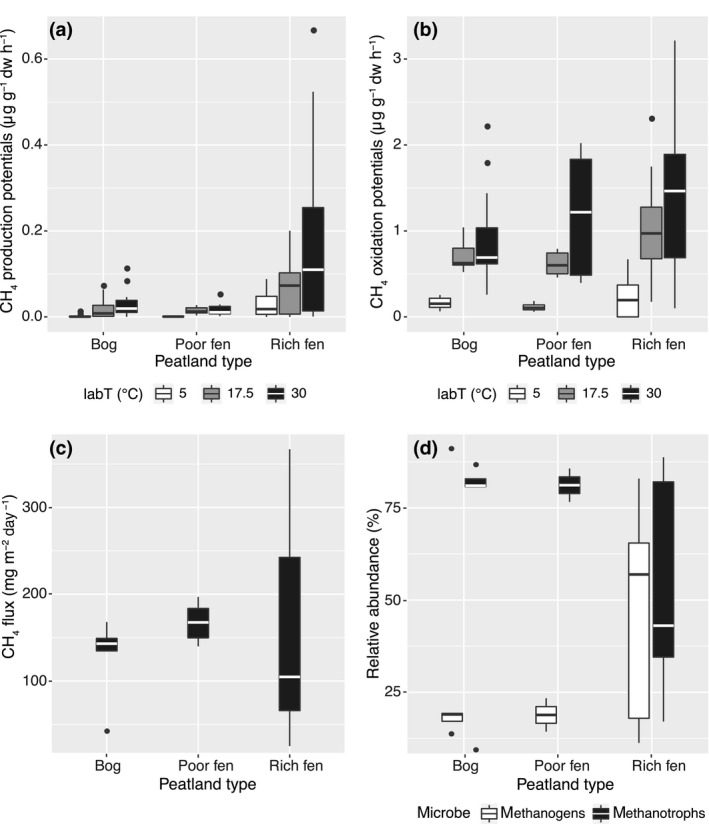
(a) Methane (CH_4_) production, and (b) oxidation potential rates at laboratory temperatures (labT) 5, 17.5, and 30℃ for the three peatland types. (c) July–August 2015 CH_4_ flux values for the three peatland types. Flux data were collected by previous studies from lawn surfaces at the same peatland sites, although the measurement points and timings were different from our laboratory incubation samples. (d) The relative abundance of CH_4_‐related microbial communities, that is, methanotrophs and methanogens, for the three peatland types

As demonstrated in the DCA and PCA analyses, the study sites were distinctively grouped into bogs, poor fens and rich fens based on their vegetation, microbial composition, and CH_4_ production and oxidation rates (Figures 4 and 5; Figure S2). We further averaged the site‐specific CH_4_ production, oxidation, and flux data (Figure S4) for these three peatland types (Figure 6). CH_4_ oxidation showed more variation within the site‐types (large range in the box plots) than CH_4_ production (Figure 6). For production, bogs and poor fens yielded very small within‐peatland‐type variations, and their potential rates were similarly low (<0.1 μg CH_4_ g^−1^ dw h^−1^) at all three temperatures, while rich fens exhibited larger variations with observed rates ranging from 0 to a maximum of 0.09, 0.20, and 0.67 μg CH_4_ g^−1^ dw h^−1^ at 5, 17.5 and 30℃, respectively. Mean CH_4_ production rate in the rich fens was greater than the other peatland types at all three temperature levels, although the differences were not significant (*p* = 0.06, *df* = 11, *F* = 2.65 at 5℃, *p* = 0.09, *df* = 11, *F* = 2.26 at 17.5℃, and *p* = 0.1, *df* = 11, *F* = 1.93 at 30℃).

For oxidation, the differences between peatland types were smaller and non‐significant (min. *p* = 0.24) due to the large variability within the peatland type. For oxidation at 5℃, the rates were generally below 0.5 μg CH_4_ g^−1^ dw h^−1^, while at 17.5℃, the rates were c. 0.52–1.04 (mean = 0.70), 0.46–0.79 (0.62), and 0.18–2.30 (1.00) μg CH_4_ g^−1^ dw h^−1^ for bogs, poor fens, and rich fens, respectively. When the temperature increased to 30℃, the values were c. 0.26–2.21 (mean = 0.90), 0.40–2.02 (1.19), and 0.10–3.21 (1.36) μg CH_4_ g^−1^ dw h^−1^ for bogs, poor fens, and rich fens. Similar to CH_4_ production, greater flux values and methanogen abundance values were recorded from rich fens, which also exhibited the greatest variation in all these parameters (Figure 6c,d; Figure S5).

### 3.5 Temperature response of CH_4_ oxidation and production

For each peatland type, CH_4_ production and oxidation rates increased with increasing temperature (Figure 6a,b), with oxidation showing a more rapid increase than production.

**FIGURE 7:**
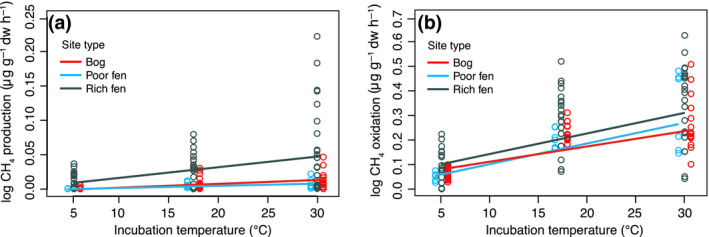
Temperature response of log‐transformed (a) methane (CH_4_) production and (b) oxidation potentials at the three peatland site types according to the mixed effects models with temperature, site type and their interaction as fixed predictors (represented by lines). Points represent the measured values. For a better visualization, the incubation temperatures were shifted +0.5 and −0.5℃ for bog and poor fen, respectively. Model parameters are presented in Table S4a

The mixed effect models that were used to test the differences in temperature responses between the peatland types (Figure 7; Table S4a) indicated that CH_4_ production in rich fens responded more steeply to higher temperatures than in bogs (*p* = 0.0003) and poor fens (*p* = 0.0028). For CH_4_ oxidation, no difference in temperature response was observed between peatland types (Figure 7; Table S4a).

**FIGURE 8:**
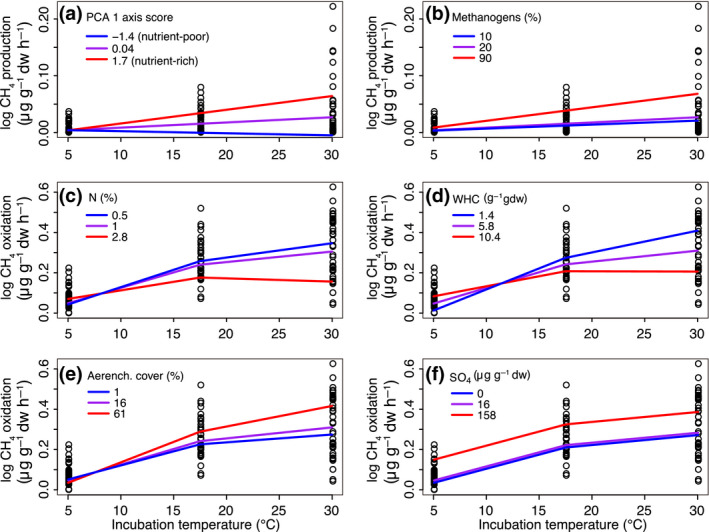
Temperature responses of methane (CH_4_) (a–b) production, and (c–f) oxidation potentials based on the mixed effects models that quantified the impact of peat properties, plant functional types, and microbial communities on these processes. Each panel represents the response to one variable so that the presented variable is allowed to vary according to the range of the data; minimum (in blue), median (in purple) and maximum (in red), while the other variables are constrained to the median value. Points represent the measured values. Model parameters are presented in Table S4b

In the mixed effect models that were used to investigate the drivers of temperature response (Table S4b), CH_4_ production potentials showed a linear response to temperature (Figure 8a,b). PCA axis 1 describes the gradient in peat property and plant functional type conditions from bogs to rich fens. It showed an interaction with temperature (*p* < 0.001); the temperature response became steeper toward the more nutrient rich sites (Figure 8a). Similarly, the temperature response of the CH_4_ production potential became steeper with increasing abundance of methanogens (*p* = 0.011 for the interaction; Figure 8b).

Oxidation potential increased with temperature, but the increase slowed down at higher temperatures; this was described in the mixed‐effects model by a second‐degree polynomial response. N content, WHC, and the cover of aerenchymous plants had an interaction with temperature (*p* < 0.001, *p* < 0.001 and *p* = 0.011, respectively; Figure 8c–e). N content and WHC flattened the temperature response of CH_4_ oxidation, while the cover of aerenchymous plants contributed to the steeper temperature response. In addition, peat SO_4_^2−^ content slightly increased CH_4_ oxidation (*p* = 0.051) without an interaction with temperature (Figure 8f).

## 4 DISCUSSION

### 4.1 Peatland type as predictor of CH_4_ production and oxidation potentials

Our results reveal the varying roles of CH_4_ production and oxidation processes in defining the rate of CH_4_ emissions. Specifically, we observed that the differences in CH_4_ emissions between peatland types (Knox et al., 2019; Moore & Knowles, 1990; Turetsky et al., 2014) are more likely caused by differences in production than in oxidation, and instead of site‐to‐site variation, oxidation seems to exhibit consistently large variations within each peatland type (e.g., larger range in the box plots in Figure 6b). We observed the greatest CH_4_ emissions, production rates and methanogen abundances in the rich fens, but simultaneously they also displayed the largest range in these variables, undermining the statistical significance. It should be noted that our sampling was conducted at all sites from a lawn habitat, which also impact the observed differences.

### 4.2 Impacts of peat properties, plant functional types and microbial communities on peatland CH_4_ production and oxidation processes

**FIGURE 9:**
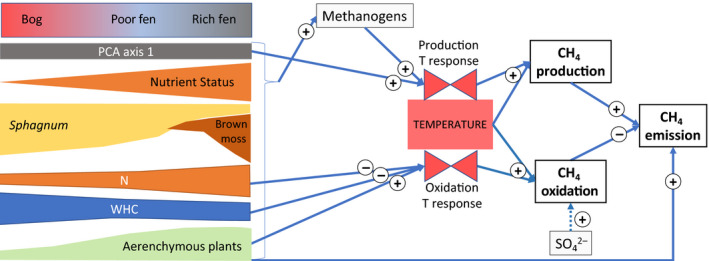
Factors affecting peatland methane (CH_4_) production and oxidation according to the results from the mixed effect models in this study, updated from Figure 1 which is literature‐based. Temperature sensitivity of production increased along PCA axis 1, which describes the main variation in site properties from bogs to rich fens (see Figure 5), and the increasing abundance of methanogens. Temperature sensitivity of oxidation increased with a greater cover of aerenchymous plants but decreased with increasing nitrogen (N) content and water holding capacity (WHC). The dashed line indicates the relationship between CH_4_ oxidation and sulfate (SO_4_^2−^), which was close to significant in the model (*p* = 0.0507)

Previous studies have shown that between‐site differences in peatland CH_4_ emissions are typically controlled by soil temperature, water table and vegetation (phenology and species composition), but have also indicated that these relationships can be modified by peatland type, region, and disturbances, such as permafrost thawing or fire (Bridgham et al., 2013; Knox et al., 2019; Turetsky et al., 2014). Our study adds to this knowledge by clarifying the different peat property and plant functional type controls on the CH_4_ production and oxidation processes (Figure 9). In our experiment, many of the studied factors impacted CH_4_ production and oxidation by affecting the temperature response of these processes, rather than increasing their overall level (Figure 9).

As we hypothesized in Figure 1 (based on earlier studies by e.g., Bergman et al., 1998; Godin et al., 2012; Juottonen et al., 2005; Yavitt et al., 1988), the CH_4_ production potential was found to be strongly related to the gradient from ombrotrophic bogs to rich fens. In our study, the position of the site within the bog‐rich fen gradient did not alter the level of CH_4_ production but increased the temperature response of the CH_4_ production potential (Figure 9). Along this gradient, nutrient availability increases and plant composition changes from *Sphagnum* dominated ombrotrophic bogs to brown moss dominated rich fens. Earlier research (Yavitt et al., 2012) supports our finding that the relative abundance of methanogens also increases along the bog‐rich fen gradient. Previously, alternative electron acceptors (e.g., sulfate, nitrate, phosphate, ammonium, nitrate) have been found to significantly reduce CH_4_ production rates as these compounds are favored in the anaerobic metabolic pathway before methanogenic conditions can be established, and their abundance may even explain the differences between the sites (e.g., Deng et al., 2017; Peters & Conrad, 1996). Interestingly, we did not find evidence that alternative electron acceptors impact CH_4_ production potential in our sites, and the variation in the amount of these substances did not show clear trends related to the bog‐rich fen gradient.

Rich fens generally exhibit greater methanogen and lower methanotroph abundances, but the microbial community also varies within site types. This was demonstrated by the methanogen abundance enhancing the CH_4_ production potential after the bog‐rich fen gradient had already been included into the model. The typically low water level conditions observed in bogs, combined with a thicker oxic zone, are associated with the dominance of plants that produce recalcitrant litter (such as hummock Sphagna and woody vascular plant species), which is considered sub‐optimal for the development of an active community of methanogens (Valentine et al., 1994). A shift in vegetation toward woody plants with recalcitrant litter may also occur in fen sites after water level drawdown (Kokkonen et al., 2019; Mäkiranta et al., 2018; Strakova et al., 2012), creating a hostile environment for methanogens (Peltoniemi et al., 2016; Yrjälä et al., 2011). While our sampling was directed at surfaces with a similar water level (at 0–5 cm below the surface) at all sites, bogs still appeared to have lower CH_4_ production values and relative abundance of methanogens than rich fens. Therefore, the nutrient level and the associated differences in vegetation along the bog‐rich fen gradient could be stronger factors in driving peatland CH_4_ production than water level.

In contrast to our hypothesis (Figure 1) and previous literature (e.g., Larmola et al., 2010; Putkinen et al., 2018; Yrjälä et al., 2011), CH_4_ oxidation potential was not found to differ between peatland site types or to be dependent on *Sphagnum* cover and methanotroph abundance. In the mixed‐effects model, oxidation potential exhibited a nonlinear response to temperature, stabilizing mildly at higher temperatures. This temperature response was negatively related to N content and the WHC of the peat, but positively related to aerenchymous species cover (Figure 9). Both WHC and aerenchymous species cover were indirectly linked to the availability of oxygen in the peat with aerenchymous plants facilitating the transport of oxygen and CH_4_ with their specialized tissue (Fritz et al., 2011; Greenup et al., 2000), while greater WHC in the peat hinders oxygen availability. The evidence on how N content affects CH_4_ oxidation is controversial; oxidation in pristine ecosystems has been suggested to be N limited (Bodelier & Laanbroek, 2004), although the addition of N (as NH_4_^+^) has been widely reported to hamper CH_4_ oxidation (Bosse et al., 1993; Conrad & Rothfuss, 1991; Hester et al., 2018). In bog sites and non‐wetland soils, N addition has been reported to stimulate CH_4_ oxidation at low concentrations but shows an inhibiting effect at higher concentrations (Aronson & Helliker, 2010; Keller et al., 2006). The inhibitory effect of high N levels is in line with our results, where N did not impact the level of oxidation but decreased its temperature response at high N contents. Finally, we observed a slight enhancing effect of SO_4_^2−^ on CH_4_ oxidation, which was on the border of statistical significance in our model. SO_4_^2−^ acts as an alternative electron acceptor not only for CH_4_ production, but also in anaerobic CH_4_ oxidation (AOM) in marine and freshwater environments (Eller et al., 2005; Schubert et al., 2011; Valentine, 2002). This process has also been found to be widespread across peatland types, although the alternative electron acceptors in peatlands remain uncertain (Gupta et al., 2013; Miller et al., 2019). Our result could be explained by anoxic microsites that remained within the peat sample in the oxic incubations, as suggested by the initial increase in CH_4_ concentration in some of the oxic incubation bottles (Figure S1). While only the linearly decreasing part of these incubations was used in the further analysis, the microsites could also have acted as sites of AOM in the presence of the electron acceptor, adding to the level of CH_4_ oxidation.

### 4.3 Temperature response of CH_4_ production and oxidation

Our results suggest that soil warming will increase both CH_4_ production and oxidation activities in all peatland types. Observed CH_4_ production in rich fens displayed a steeper temperature response than bogs and poor fens, which is in contrast with previous studies that used Q_10_ values as a parameter of temperature sensitivity (Bergman et al., 1998, 2000; Lupascu et al., 2012). In these studies (and also in our own dataset), very little CH_4_ (close to zero) is produced in *Sphagnum*‐dominated peat at low temperatures, so that even a small increase in production at higher temperatures can lead to very large Q_10_ values (i.e., higher sensitivity). In comparison to CH_4_ production, the rate of CH_4_ oxidation, in general, showed a steeper response to warmer temperatures in all peatland types. The temperature response of oxidation was similar between peatland types, which may have been caused by the detected drivers of the temperature response counterbalancing each other within the fen‐bog gradient, that is, high N content and low WHC that characterize rich fens decreased the temperature response, while high aerenchymous plant cover led to an increase. We assume that the impact of these environmental variables on temperature response is due to the availability of a substrate that limits the extent to which microbes are able to take advantage of the rising temperature. In the case of WHC, elevated levels would limit the availability of oxygen, and microbial oxidation would increase less steeply with rising temperatures. Nitrogen, in turn, may limit the substrate for CH_4_ oxidation through substrate competition (Bosse et al., 1993; Conrad & Rothfuss, 1991; Hester et al., 2018). The steeper temperature response of oxidation compared to production has been previously found in other ecosystems, for example, lakes (Fuchs et al., 2016; Lofton et al., 2014), which suggests that oxidation could better offset increased CH_4_ production with increasing temperature. Based on our findings, this offset could be stronger in bogs and poor fens where the temperature response of production was milder.

One of the possible limitations of our approach, whereby we added a fixed CH_4_ concentration to all the oxidation potential incubation bottles from the different sites, is that the actual concentrations in the peat may vary between site types and, over time, may affect the oxidation potential rates. We selected the initial concentration (5000 ppm) based on previous direct measurements of CH_4_ concentrations in the peat at three of the studied sites, which showed mean growing season concentrations of up to 14,000 ppm at the maximum depth of our sampling. A more correct, but laborious approach, would have been to pre‐sample below‐surface CH_4_ concentrations at each site prior to the experiment to determine the correct site‐specific starting concentration for the oxidation potential measurements. It is important to bear in mind that as our results are based on simple sampling conducted at the peak of the growing season, they also represent the maximum potentials. Another important issue is the 10‐day incubation time in the CH_4_ production potential measurements, which might be too short in light of previous results from permafrost soils or peat samples collected in winter (Gao et al., 2019; Treat et al., 2015). Indeed, in permafrost and mineral soils where the methanogens are likely to be dormant, longer incubation times are essential. It is evident from previous studies (Juottonen et al., 2008; Putkinen et al., 2018; Saarnio et al., 1997) and from this study (Figure S1) that production starts almost immediately in an active peat layer in the peak season and that shorter incubation periods of up to 1–2 weeks are feasible. In this study, we did not attempt to disentangle all the processes that impact CH_4_ production. Instead, our objective was to measure the production potential as a net result of the biogeochemical environment at the sites, including the alternative electron acceptors, as well as the different CH_4_ production pathways (Deng et al., 2017). However, we assume that the measured production potential is realistic in relation to field conditions, for example, in the case of alternative electron acceptors that suppress CH_4_ production rates at the sites, their abundance would not change during the sample transportation and the laboratory result would thus reflect the real conditions.

Altogether, our results imply that warming could increase the differences in the balance of CH_4_ oxidation and production between peatland types by increasing the production potential at brown moss‐dominated rich fens more than in *Sphagnum*‐dominated poor fens and bogs, with a more uniform impact on oxidation. In our study, the CH_4_ fluxes measured in the field were not correlated with the potential CH_4_ production and oxidation. This suggests that the prediction of CH_4_ emissions also requires knowledge of the processes beyond CH_4_ production and oxidation, such as CH_4_ transport by aerenchymous plants. In future studies, however, in‐situ flux measurements should be carried out at the specific points at the same time when samples are collected for laboratory incubation. Also, the incubation conditions should be as similar as possible to the *in situ* field conditions, which might help to better bind together the mechanisms behind CH_4_ emissions. We acknowledge that only lawn habitats were investigated in this study and that we were not able to consider the thermal adaptation of microbes, such as the possible increase in methanogen abundance with warming (Turetsky et al., 2008), and that the situation is more complicated under real field conditions at the whole‐ecosystem level.

Although we observed that both production and oxidation increase with warmer temperatures, the net flux may still not increase accordingly, as other factors can influence CH_4_ dynamics. The impact of a warmer climate on vegetation dynamics, for example, can counteract part of the direct response. Future CH_4_ emissions under a warming climate are strongly dependent on the successional development of peatland vegetation. The scenario of *Sphagnum* invasion under a warming climate (Magnan et al., 2018; Tahvanainen, 2011) and a development toward *Sphagnum*‐dominated peatlands (Fritz et al., 2014; Tuittila et al., 2013) is likely to mitigate the increasing rate of CH_4_ emissions under a warming climate, while the increased areal cover of wet rich fens due to permafrost thawing is likely to lead to greater CH_4_ emissions. In recent years, global CH_4_ modeling has made progress by the inclusion of microbial processes into the peatland models (see Chadburn et al., 2020; Nzotungicimpaye et al., 2020). Our results highlight the importance of this model development, as methanogen and methanotroph dynamics appear to have distinct sets of environmental drivers.

## CONFLICT OF INTEREST

The authors declare that they have no conflict of interest.

## AUTHOR CONTRIBUTIONS

The study was designed by AL, EST, AK, and NW in a *Finnish peatlanders* meeting in Koli. SU and MM collected samples and conducted field measurements. NW and JK performed the laboratory incubation and gas concentration measurements. DE conducted DNA analysis and sequencing. HZ, AK, AML, EST, and AL performed the data analyses. HZ led the writing of the manuscript. All other authors contributed through the collection of samples, and/or discussions and comments on the text.

## Supporting information

Supplementary Material

## Data Availability

The DNA sequences that support the findings of this study are openly available in the NCBI Sequence Read Archive under project accession number PRJNA679629. The other data used in this study are available upon reasonable request from the corresponding author.

## References

[gcb15740-bib-0001] Aerts, R. , Verhoeven, J. T. A. , & Whigham, D. F. (1999). Plant‐mediated controls on nutrient cycling in temperate fens and bogs. Ecology, 80(7), 2170–2181.

[gcb15740-bib-0002] Aronson, E. L. , & Helliker, B. R. (2010). Methane flux in non‐wetland soils in response to nitrogen addition: A meta‐analysis. Ecology, 91(11), 3242–3251. 10.1890/09-2185.1 21141185

[gcb15740-bib-0003] Bergman, I. , Klarqvist, M. , & Nilsson, M. (2000). Seasonal variation in rates of methane production from peat of various botanical origins: Effects of temperature and substrate quality. Fems Microbiology Ecology, 33(3), 181–189. 10.1016/S0168-6496(00)00060-X 11098069

[gcb15740-bib-0004] Bergman, I. , Svensson, B. H. , & Nilsson, M. (1998). Regulation of methane production in a Swedish acid mire by pH, temperature and substrate. Soil Biology & Biochemistry, 30(6), 729–741. 10.1016/S0038-0717(97)00181-8

[gcb15740-bib-0005] Blunier, T. , Chappellaz, J. , Schwander, J. , Stauffer, B. , & Raynaud, D. (1995). Variations in atmospheric methane concentration during the Holocene Epoch. Nature, 374(6517), 46–49. 10.1038/374046a0

[gcb15740-bib-0006] Bodelier, P. L. E. , & Laanbroek, H. J. (2004). Nitrogen as a regulatory factor of methane oxidation in soils and sediments. Fems Microbiology Ecology, 47(3), 265–277. 10.1016/s0168-6496(03)00304-0 19712315

[gcb15740-bib-0007] Bosse, U. , Frenzel, P. , & Conrad, R. (1993). Inhibition of methane oxidation by ammonium in the surface layer of a littoral sediment. Microbiology Ecology, 13(2), 123–134. 10.1111/j.1574-6941.1993.tb00058.x

[gcb15740-bib-0008] Bousquet, P. , Ringeval, B. , Pison, I. , Dlugokencky, E. J. , Brunke, E. G. , Carouge, C. , Chevallier, F. , Fortems‐Cheiney, A. , Frankenberg, C. , Hauglustaine, D. A. , Krummel, P. B. , Langenfelds, R. L. , Ramonet, M. , Schmidt, M. , Steele, L. P. , Szopa, S. , Yver, C. , Viovy, N. , & Ciais, P. (2011). Source attribution of the changes in atmospheric methane for 2006–2008. Atmospheric Chemistry and Physics, 11(8), 3689–3700. 10.5194/acp-11-3689-2011

[gcb15740-bib-0009] Bridgham, S. D. , Cadillo‐Quiroz, H. , Keller, J. K. , & Zhuang, Q. L. (2013). Methane emissions from wetlands: Biogeochemical, microbial, and modeling perspectives from local to global scales. Global Change Biology, 19(5), 1325–1346. 10.1111/gcb.12131 23505021

[gcb15740-bib-0010] Brook, E. J. , Harder, S. , Severinghaus, J. , Steig, E. J. , & Sucher, C. M. (2000). On the origin and timing of rapid changes in atmospheric methane during the last glacial period. Global Biogeochemical Cycles, 14(2), 559–572. 10.1029/1999gb001182

[gcb15740-bib-0011] Çamdevýren, H. , Demýr, N. , Kanik, A. , & Keskýn, S. (2005). Use of principal component scores in multiple linear regression models for prediction of Chlorophyll‐a in reservoirs. Ecological Modelling, 181(4), 581–589. 10.1016/j.ecolmodel.2004.06.043

[gcb15740-bib-0012] Chadburn, S. E. , Aalto, T. , Aurela, M. , Baldocchi, D. , Biasi, C. , Boike, J. , Burke, E. J. , Comyn‐Platt, E. , Dolman, A. J. , Duran‐Rojas, C. , Fan, Y. , Friborg, T. , Gao, Y. , Gedney, N. , Göckede, M. , Hayman, G. D. , Holl, D. , Hugelius, G. , Kutzbach, L. , … Westermann, S. (2020). Modeled microbial dynamics explain the apparent temperature‐sensitivity of wetland methane emissions. Global Biogeochemical Cycles, 34(11). 10.1029/2020GB006678

[gcb15740-bib-0013] Chappellaz, J. , Blunier, T. , Raynaud, D. , Barnola, J. M. , Schwander, J. , & Stauffer, B. (1993). Synchronous changes in atmospheric CH_4_ and Greenland climate between 40‐Kyr and 8‐Kyr BP. Nature, 366(6454), 443–445. 10.1038/366443a0

[gcb15740-bib-0014] Charman, D. J. (2007). Summer water deficit variability controls on peatland water‐table changes: Implications for Holocene palaeoclimate reconstructions. The Holocene, 17(2), 217–227. 10.1177/0959683607075836

[gcb15740-bib-0015] Christensen, T. R. , Ekberg, A. , Strom, L. , Mastepanov, M. , Panikov, N. , Oquist, M. , Svensson, B. H. , Nykanen, H. , Martikainen, P. J. , & Oskarsson, H. (2003). Factors controlling large scale variations in methane emissions from wetlands. Geophysical Research Letters, 30(7), 10.1029/2002gl016848

[gcb15740-bib-0016] Ciais, P. , Sabine, C. , Bala, G. , Bopp, L. , Brovkin, V. , Canadell, J. , Chhabra, A. , DeFries, R. , Galloway, J. , Heimann, M. , Jones, C. , Le Quéré, C. , Myneni, R. B. , Piao, S. , & Thornton, P. (2013). Carbon and other biogeochemical cycles. In T. F. Stocker , D. Qin , G.‐K. Plattner , M. Tignor , S. K. Allen , J. Boschung , A. Nauels , Y. Xia , V. Bex , & P. M. Midgley (Eds.), Climate change 2013: The physical science basis. Contribution of working group I to the fifth assessment report of the intergovernmental panel on climate change. Cambridge University Press.

[gcb15740-bib-0017] Conrad, R. , & Rothfuss, F. (1991). Methane oxidation in soil surface layer of a flooded rice field and the effect of ammonium. Biology and Fertility of Soils, 12, 28–32. 10.1007/BF00369384

[gcb15740-bib-0018] Deng, J. , McCalley, C. K. , Frolking, S. , Chanton, J. , Crill, P. , Varner, R. , Tyson, G. , Rich, V. , Hines, M. , Saleska, S. R. , & Li, C. (2017). Adding stable carbon isotopes improves model representation of the role of microbial communities in peatland methane cycling. Journal of Advances in Modeling Earth Systems, 9(2), 1412–1430. 10.1002/2016MS000817

[gcb15740-bib-0019] Dlugokencky, E. J. , Nisbet, E. G. , Fisher, R. , & Lowry, D. (2011). Global atmospheric methane: Budget, changes and dangers. Philosophical Transactions of the Royal Society A: Mathematical, Physical and Engineering Sciences, 369(1943), 2058–2072. 10.1098/rsta.2010.0341 21502176

[gcb15740-bib-0020] Eller, G. , Känel, L. , & Krüger, M. (2005). Cooccurrence of aerobic and anaerobic methane oxidation in the water column of lake Plußsee. Applied and Environmental Microbiology, 71, 8925–8928. 10.1128/AEM.71.12.8925-8928.2005 16332891 PMC1317442

[gcb15740-bib-0021] Fawcett, J. K. , & Scott, J. E. (1960). A rapid and precise method for the determination of urea. Journal of Clinical Pathology, 13(2), 156–159. 10.1136/jcp.13.2.156 13821779 PMC480024

[gcb15740-bib-0022] Fletcher, S. E. M. , Tans, P. P. , Bruhwiler, L. M. , Miller, J. B. , & Heimann, M. (2004). CH_4_ sources estimated from atmospheric observations of CH_4_ and its ^13^C/^12^C isotopic ratios: 1. Inverse modeling of source processes. Global Biogeochemical Cycles, 18(4). 10.1029/2004gb002223

[gcb15740-bib-0023] Fritz, C. , Lamers, L. P. M. , Riaz, M. , van den Berg, L. J. L. , & Elzenga, T. J. T. M. (2014). Sphagnum mosses – Masters of efficient N‐uptake while avoiding intoxication. PLoS One, 9(1), e79991. 10.1371/journal.pone.0079991 24416125 PMC3886977

[gcb15740-bib-0024] Fritz, C. , Pancotto, V. A. , Elzenga, J. T. M. , Visser, E. J. W. , Grootjans, A. P. , Pol, A. , Iturraspe, R. , Roelofs, J. G. M. , & Smolders, A. J. P. (2011). Zero methane emission bogs: Extreme rhizosphere oxygenation by cushion plants in Patagonia. New Phytologist, 190, 398–408. 10.1111/j.1469-8137.2010.03604.x 21232058

[gcb15740-bib-0025] Frolking, S. , & Roulet, N. T. (2007). Holocene radiative forcing impact of northern peatland carbon accumulation and methane emissions. Global Change Biology, 13(5), 1079–1088. 10.1111/j.1365-2486.2007.01339.x

[gcb15740-bib-0026] Fuchs, A. , Lyautey, E. , Montuelle, B. , & Casper, P. (2016). Effects of increasing temperatures on methane concentrations and methanogenesis during experimental incubation of sediments from oligotrophic and mesotrophic lakes. Journal of Geophysical Research‐Biogeosciences, 121(5), 1394–1406. 10.1002/2016jg003328

[gcb15740-bib-0027] Gao, C. , Sander, M. , Agethen, S. , & Knorr, K.‐H. (2019). Electron accepting capacity of dissolved and particulate organic matter control CO_2_ and CH_4_ formation in peat soils. Geochimica et Cosmochimica Acta, 245, 266–277. 10.1016/j.gca.2018.11.004

[gcb15740-bib-0028] Godin, A. , McLaughlin, J. W. , Webster, K. L. , Packalen, M. , & Basiliko, N. (2012). Methane and methanogen community dynamics across a boreal peatland nutrient gradient. Soil Biology and Biochemistry, 48, 96–105. 10.1016/j.soilbio.2012.01.018

[gcb15740-bib-0029] Gorham, E. (1991). Northern peatlands – Role in the carbon‐cycle and probable responses to climatic warming. Ecological Applications, 1(2), 182–195. 10.2307/1941811 27755660

[gcb15740-bib-0030] Greenup, A. L. , Bradford, M. A. , McNamara, N. P. , Ineson, P. , & Lee, J. A. (2000). The role of *Eriophorum vaginatum* in CH_4_ flux from an ombrotrophic peatland. Plant and Soil, 227(1–2), 265–272. 10.1023/A:1026573727311

[gcb15740-bib-0031] Gupta, V. , Smemo, K. A. , Yavitt, J. B. , Fowler, D. , Branfireun, B. , & Basiliko, N. (2013). Stable isotopes reveal widespread anaerobic methane oxidation across latitude and peatland type. Environmental Science & Technology, 47(15), 8273–8279. 10.1021/es400484t 23822884

[gcb15740-bib-0032] Helbig, M. , Waddington, J. M. , Alekseychik, P. , Amiro, B. D. , Aurela, M. , Barr, A. G. , Black, T. A. , Blanken, P. D. , Carey, S. K. , Chen, J. , Chi, J. , Desai, A. R. , Dunn, A. , Euskirchen, E. S. , Flanagan, L. B. , Forbrich, I. , Friborg, T. , Grelle, A. , Harder, S. , … Zyrianov, V. (2020). Increasing contribution of peatlands to boreal evapotranspiration in a warming climate. Nature Climate Change, 10(6), 555–560. 10.1038/s41558-020-0763-7

[gcb15740-bib-0033] Hester, E. R. , Harpenslager, S. F. , van Diggelen, J. , Lamers, L. L. , Jetten, M. , Lüke, C. , Lucker, S. , & Welte, C. U. (2018). Linking nitrogen load to the structure and function of wetland soil and rhizosphere microbial communities. Ecological and Evolutionary Science, 3(1), e00214–e00217. 10.1128/mSystems.00214-17 PMC5790874 29404427

[gcb15740-bib-0034] Hopple, A. M. , Wilson, R. M. , Kolton, M. , Zalman, C. A. , Chanton, J. P. , Kostka, J. , Hanson, P. J. , Keller, J. K. , & Bridgham, S. D. (2020). Massive peatland carbon banks vulnerable to rising temperatures. Nature Communications, 11(1), 1–7. 10.1038/s41467-020-16311-8 PMC7217827 32398638

[gcb15740-bib-0035] IPCC . (2013). Climate change 2013: The physical science basis. In T. F. Stocker , D. Qin , G.‐K. Plattner , M. Tignor , S. K. Allen , J. Boschung , A. Nauels , Y. Xia , V. Bex , & P. M. Midgley (Eds.), Contribution of working group I to the fifth assessment report of the intergovernmental panel on climate change. Cambridge University Press, 1535pp.

[gcb15740-bib-0036] Jaatinen, K. , Fritze, H. , Laine, J. , & Laiho, R. (2007). Effects of short‐ and long‐term water‐level drawdown on the populations and activity of aerobic decomposers in a boreal peatland. Global Change Biology, 13(2), 491–510. 10.1111/j.1365-2486.2006.01312.x

[gcb15740-bib-0037] Jaatinen, K. , Tuittila, E. S. , Laine, J. , Yrjala, K. , & Fritze, H. (2005). Methane‐oxidizing bacteria in a Finnish raised mire complex: Effects of site fertility and drainage. Microbial Ecology, 50(3), 429–439. 10.1007/s00248-004-0219-z 16283115

[gcb15740-bib-0038] Joabsson, A. , Christensen, T. R. , & Wallen, B. (1999). Vascular plant controls on methane emissions from northern peat‐forming wetlands. Trends in Ecology & Evolution, 14(10), 385–388. 10.1016/S0169-5347(99)01649-3 10481199

[gcb15740-bib-0039] Juottonen, H. , Galand, P. E. , Tuittila, E.‐S. , Laine, J. , Fritze, H. , & Yrjala, K. (2005). Methanogen communities and bacteria along an ecohydrological gradient in a northern raised bog complex. Environmental Microbiology, 7(10), 1547–1557. 10.1111/j.14622920.2005.00838.x 16156728

[gcb15740-bib-0040] Juottonen, H. , Tuittila, E.‐S. , Juutinen, S. , Fritze, H. , & Yrjälä, K. (2008). Seasonality of rDNA‐ and rRNA‐derived archaeal communities and methanogenic potential in a boreal mire. The ISME Journal, 2, 1157–1168. 10.1038/ismej.2008.66 18650929

[gcb15740-bib-0041] Keller, J. K. , Bauers, A. K. , Bridgham, S. D. , Kellogg, L. E. , & Iversen, C. M. (2006). Nutrient control of microbial carbon cycling along an ombrotrophic‐minerotrophic peatland gradient. Journal of Geophysical Research: Biogeosciences, 111(G3). 10.1029/2005JG000152

[gcb15740-bib-0042] Kettunen, A. , Kaitala, V. , Lehtinen, A. , Lohila, A. , Alm, J. , Silvola, J. , & Martikainen, P. J. (1999). Methane production and oxidation potentials in relation to water table fluctuations in two boreal mires. Soil Biology & Biochemistry, 31(12), 1741–1749. 10.1016/S0038-0717(99)00093-0

[gcb15740-bib-0043] Kirschke, S. , Bousquet, P. , Ciais, P. , Saunois, M. , Canadell, J. G. , Dlugokencky, E. J. , Bergamaschi, P. , Bergmann, D. , Blake, D. R. , Bruhwiler, L. , Cameron‐Smith, P. , Castaldi, S. , Chevallier, F. , Feng, L. , Fraser, A. , Heimann, M. , Hodson, E. L. , Houweling, S. , Josse, B. , … Zeng, G. (2013). Three decades of global methane sources and sinks. Nature Geoscience, 6(10), 813–823. 10.1038/Ngeo1955

[gcb15740-bib-0044] Knox, S. H. , Jackson, R. B. , Poulter, B. , McNicol, G. , Fluet‐Chouinard, E. , Zhang, Z. , Hugelius, G. , Bousquet, P. , Canadell, J. G. , Saunois, M. , Papale, D. , Chu, H. , Keenan, T. F. , Baldocchi, D. , Torn, M. S. , Mammarella, I. , Trotta, C. , Aurela, M. , Bohrer, G. , … Zona, D. (2019). FLUXNET‐CH_4_ synthesis activity: Objectives, observations, and future directions. Bulletin of the American Meteorological Society, 100(12), 2607–2632. 10.1175/Bams-D-18-0268.1

[gcb15740-bib-0045] Kokkonen, N. A. K. , Laine, A. M. , Laine, J. , Vasander, H. , Kurki, K. , Gong, J. , & Tuittila, E.‐S. (2019). Responses of peatland vegetation to 15‐year water level drawdown as mediated by fertility level. Journal of Vegetation Science, 30(6), 1206–1216. 10.1111/jvs.12794

[gcb15740-bib-0046] Korhola, A. , Ruppel, M. , Seppä, H. , Väliranta, M. , Virtanen, T. , & Weckström, J. (2010). The importance of northern peatland expansion to the late‐Holocene rise of atmospheric methane. Quaternary Science Reviews, 29(5–6), 611–617. 10.1016/j.quascirev.2009.12.010

[gcb15740-bib-0047] Laine, A. M. , Mehtätalo, L. , Tolvanen, A. , Frolking, S. , & Tuittila, E.‐S. (2019). Combined effect of drainage, restoration and warming on boreal mire greenhouse gas fluxes. Science of the Total Environment, 647, 169–181. 10.1016/j.scitotenv.2018.07.390 30077847

[gcb15740-bib-0048] Larmola, T. , Tuittila, E. S. , Tiirola, M. , Nykanen, H. , Martikainen, P. J. , Yrjala, K. , Tuomivirta, T. , & Fritze, H. (2010). The role of *Sphagnum* mosses in the methane cycling of a boreal mire. Ecology, 91(8), 2356–2365. 10.1890/09-1343.1 20836457

[gcb15740-bib-0049] Lofton, D. D. , Whalen, S. C. , & Hershey, A. E. (2014). Effect of temperature on methane dynamics and evaluation of methane oxidation kinetics in shallow Arctic Alaskan lakes. Hydrobiologia, 721(1), 209–222. 10.1007/s10750-013-1663-x

[gcb15740-bib-0050] Lupascu, M. , Wadham, J. L. , Hornibrook, E. R. C. , & Pancost, R. D. (2012). Temperature sensitivity of methane production in the permafrost active layer at Stordalen, Sweden: A comparison with non‐permafrost Northern wetlands. Arctic, Antarctic, and Alpine Research, 44(4), 469–482. 10.1657/1938-4246-44.4.469

[gcb15740-bib-0051] MacDonald, G. M. , Beilman, D. W. , Kremenetski, K. V. , Sheng, Y. W. , Smith, L. C. , & Velichko, A. A. (2006). Rapid early development of circumarctic peatlands and atmospheric CH_4_ and CO_2_ variations. Science, 314(5797), 285–288. 10.1126/science.1131722 17038618

[gcb15740-bib-0052] Magnan, G. , van Bellen, S. , Davies, L. , Froese, D. , Garneau, M. , Mullan‐Boudreau, G. , Zaccone, C. , & Shotyk, W. (2018). Impact of the Little Ice Age cooling and 20th century climate change on peatland vegetation dynamics in central and northern Alberta using a multi‐proxy approach and high‐resolution peat chronologies. Quaternary Science Reviews, 185, 230–243. 10.1016/j.quascirev.2018.01.015

[gcb15740-bib-0053] Mäkiranta, P. , Laiho, R. , Mehtötalo, L. , Strakova, P. , Sormunen, J. , Minkkinen, K. , Penttila, T. , Fritze, H. , & Tuittila, E. S. (2018). Responses of phenology and biomass production of boreal fens to climate warming under different water‐table level regimes. Global Change Biology, 24(3), 944–956. 10.1111/gcb.13934 28994163

[gcb15740-bib-0054] Mastepanov, M. , Sigsgaard, C. , Mastepanov, M. , Strom, L. , Tamstorf, M. P. , Lund, M. , & Christensen, T. R. (2013). Revisiting factors controlling methane emissions from high‐Arctic tundra. Biogeosciences, 10(7), 5139–5158. 10.5194/bg-10-5139-2013

[gcb15740-bib-0055] Miller, K. E. , Lai, C.‐T. , Dahlgren, R. A. , & Lipson, D. A. (2019). Anaerobic methane oxidation in high‐Arctic Alaskan peatlands as a significant control on net CH_4_ fluxes. Soil Systems, 3(1), 2571–8789. 10.3390/soilsystems3010007

[gcb15740-bib-0056] Moore, T. R. , & Knowles, R. (1990). Methane emissions from fen, bog and swamp peatlands in Quebec. Biogeochemistry, 11(1), 45–61. 10.1007/BF00000851

[gcb15740-bib-0057] Nazaries, L. , Murrell, J. C. , Millard, P. , Baggs, L. , & Singh, B. K. (2013). Methane, microbes and models: Fundamental understanding of the soil methane cycle for future predictions. Environmental Microbiology, 15(9), 2395–2417. 10.1111/1462-2920.12149 23718889

[gcb15740-bib-0058] Nisbet, E. G. , Manning, M. R. , Dlugokencky, E. J. , Fisher, R. E. , Lowry, D. , Michel, S. E. , Myhre, C. L. , Platt, S. M. , Allen, G. , Bousquet, P. , Brownlow, R. , Cain, M. , France, J. L. , Hermansen, O. , Hossaini, R. , Jones, A. E. , Levin, I. , Manning, A. C. , Myhre, G. , … White, J. W. C. (2019). Very strong atmospheric methane growth in the 4 Years 2014–2017: Implications for the Paris Agreement. Global Biogeochemical Cycles, 33(3), 318–342. 10.1029/2018gb006009

[gcb15740-bib-0059] Noyce, G. L. , Varner, R. K. , Bubier, J. L. , & Frolking, S. (2014). Effect of *Carex rostrata* on seasonal and interannual variability in peatland methane emissions. Journal of Geophysical Research‐Biogeosciences, 119(1), 24–34. 10.1002/2013jg002474

[gcb15740-bib-0060] Nzotungicimpaye, C.‐M. , MacDougall, A. H. , Melton, J. R. , Treat, C. C. , Eby, M. , Lesack, L. F. W. , & Zickfeld, K. (2020). WETMETH 1.0: A new wetland methane model for implementation in Earth system models. Geoscientific Model Development Discussion (preprint). 10.5194/gmd-2020-176

[gcb15740-bib-0061] Peltoniemi, K. , Laiho, R. , Juottonen, H. , Bodrossy, L. , Kell, D. K. , Minkkinen, K. , Makiranta, P. , Mehtatalo, L. , Penttila, T. , Siljanen, H. M. P. , Tuittila, E.‐S. , Tuomivirta, T. , & Fritze, H. (2016). Responses of methanogenic and methanotrophic communities to warming in varying moisture regimes of two boreal fens. Soil Biology and Biochemistry, 97, 144–156. 10.1016/j.soilbio.2016.03.007

[gcb15740-bib-0062] Peters, V. , & Conrad, R. (1996). Sequential reduction processes and initiation of CH_4_ production upon flooding of oxic upland soils. Soil Biology and Biochemistry, 28, 371–382. 10.1016/0038-0717(95)00146-8

[gcb15740-bib-0063] Pirinen, P. , Simola, H. , Aalto, J. , Kaukoranta, J.‐P. , Karlsson, P. , & Ruuhela, R. (2012). Finnish Meteorological Institute reports. Tilastoja Suomen Ilmastosta 1981‐2010 (Climatological Statistics of Finland 1981‐2010), Vol. 1.

[gcb15740-bib-0064] Putkinen, A. , Tuittila, E.‐S. , Siljanen, H. , Bodrossy, L. , & Fritze, H. (2018). Recovery of methane turnover and the associated microbial communities in restored cutover peatlands is strongly linked with increasing Sphagnum abundance. Soil Biology and Biochemistry, 116, 110–119. 10.1016/j.soilbio.2017.10.005

[gcb15740-bib-0065] Pypker, T. G. , Moore, P. A. , Waddington, J. M. , Hribljan, J. A. , & Chimner, R. C. (2013). Shifting environmental controls on CH_4_ fluxes in a sub‐boreal peatland. Biogeosciences, 10(12), 7971–7981. 10.5194/bg-10-7971-2013

[gcb15740-bib-0066] R Core Team . (2019). R: A language and environment for statistical computing. R Foundation for Statistical Computing.

[gcb15740-bib-0067] Rigby, M. , Prinn, R. G. , Fraser, P. J. , Simmonds, P. G. , Langenfelds, R. L. , Huang, J. , Cunnold, D. M. , Steele, L. P. , Krummel, P. B. , Weiss, R. F. , O’Doherty, S. , Salameh, P. K. , Wang, H. J. , Harth, C. M. , Muhle, J. , & Porter, L. W. (2008). Renewed growth of atmospheric methane. Geophysical Research Letters, 35(22). 10.1029/2008gl036037

[gcb15740-bib-0068] Saarnio, S. , Alm, J. , Silvola, J. , Lohila, A. , Nykänen, H. , & Martikainen, P. J. (1997). Seasonal variations in CH_4_ emissions and production and oxidation potentials at microsites on an oligotrophic pine fen. Oecologia, 110, 414–422. 10.1007/s004420050176 28307231

[gcb15740-bib-0069] Schubert, C. J. , Vazquez, F. , Lösekann‐Behrens, T. , Knittel, K. , Tonolla, M. , & Boetius, A. (2011). Evidence for anaerobic oxidation of methane in sediments of a freshwater system (Lago di Cadagno). Fems Microbiology Ecology, 76(1), 26–38. 10.1111/j.1574-6941.2010.01036.x 21244447

[gcb15740-bib-0070] Sim, T. G. , Swindles, G. T. , Morris, P. J. , Baird, A. J. , Cooper, C. L. , Gallego‐Sala, A. , Charman, D. J. , Roland, T. P. , Borken, W. , Mullan, D. , Aquino‐Lopez, M. A. , & Galka, M. (2021). Divergent responses of permafrost peatlands to recent climate change. Environmental Research Letters, 16, 034001. 10.1088/1748-9326/abe00b

[gcb15740-bib-0071] Strakova, P. , Penttila, T. , Laine, J. , & Laiho, R. (2012). Disentangling direct and indirect effects of water table drawdown on above‐ and belowground plant litter decomposition: Consequences for accumulation of organic matter in boreal peatlands. Global Change Biology, 18(1), 322–335. 10.1111/j.1365-2486.2011.02503.x

[gcb15740-bib-0072] Ström, L. , Ekberg, A. , Mastepanov, M. , & Christensen, T. R. (2003). The effect of vascular plants on carbon turnover and methane emissions from a tundra wetland. Global Change Biology, 9(8), 1185–1192. 10.1046/j.1365-2486.2003.00655.x

[gcb15740-bib-0073] Swindles, G. T. , Morris, P. J. , Mullan, D. J. , Payne, R. J. , Roland, T. P. , Amesbury, M. J. , Lamentowicz, M. , Turner, T. E. , Gallego‐Sala, A. , Sim, T. , Barr, I. D. , Blaauw, M. , Blundell, A. , Chambers, F. M. , Charman, D. J. , Feurdean, A. , Galloway, J. M. , Gałka, M. , Green, S. M. , … Warner, B. (2019). Widespread drying of European peatlands in recent centuries. Nature Geoscience, 12, 922–928. 10.1038/s41561-019-0462-z

[gcb15740-bib-0074] Tahvanainen, T. (2011). Abrupt ombrotrophication of a boreal aapa mire triggered by hydrological disturbance in the catchment. Journal of Ecology, 99(2), 404–415. 10.1111/j.1365-2745.2010.01778.x

[gcb15740-bib-0075] ter Braak, C. J. F. , & Šmilauer, P. (2012). CANOCO reference manual and user's guide: Software for ordination. Version 5. Microcomputer Power.

[gcb15740-bib-0076] Treat, C. C. , Bloom, A. A. , & Marushchak, M. E. (2018). Non‐growing season methane emissions are a significant component of annual emissions across northern ecosystems. Global Change Biology, 24(8), 3331–3343. 10.1111/gcb.14137 29569301

[gcb15740-bib-0077] Treat, C. C. , Bubier, J. L. , Varner, R. K. , & Crill, P. M. (2007). Timescale dependence of environmental and plant‐mediated controls on CH_4_ flux in a temperate fen. Journal of Geophysical Research‐Biogeosciences, 112(G1). 10.1029/2006jg000210

[gcb15740-bib-0078] Treat, C. C. , Natali, S. M. , Ernakovich, J. , Iversen, C. M. , Lupascu, M. , McGuire, A. D. , Norby, R. J. , Chowdhury, T. R. , Richter, A. , Šantrůčková, H. , Schädel, C. , Schuur, E. A. G. , Sloan, V. L. , Turetsky, M. R. , & Waldrop, M. P. (2015). A pan‐Arctic synthesis of CH_4_ and CO_2_ production from anoxic soil incubations. Global Change Biology, 21, 2787–2803. 10.1111/gcb.12875 25620695

[gcb15740-bib-0079] Tuittila, E.‐S. , Juutinen, S. , Frolking, S. , Väliranta, M. , Laine, A. M. , Miettinen, A. , Seväkivi, M.‐L. , Quillet, A. , & Merilä, P. (2013). Wetland chronosequence as a model of peatland development: Vegetation succession, peat and carbon accumulation. The Holocene, 23, 23–33. 10.1177/0959683612450197

[gcb15740-bib-0080] Turetsky, M. R. , Kotowska, A. , Bubier, J. , Dise, N. B. , Crill, P. , Hornibrook, E. R. C. , Minkkinen, K. , Moore, T. R. , Myers‐Smith, I. H. , Nykanen, H. , Olefeldt, D. , Rinne, J. , Saarnio, S. , Shurpali, N. , Tuittila, E.‐S. , Waddington, J. M. , White, J. R. , Wickland, K. P. , & Wilmking, M. (2014). A synthesis of methane emissions from 71 northern, temperate, and subtropical wetlands. Global Change Biology, 20(7), 2183–2197. 10.1111/gcb.12580 24777536

[gcb15740-bib-0081] Turetsky, M. R. , Treat, C. C. , Waldrop, M. P. , Waddington, J. M. , Harden, J. W. , & McGuire, A. D. (2008). Short‐term response of methane fluxes and methanogen activity to water table and soil warming manipulations in an Alaskan peatland. Journal of Geophysical Research‐Biogeosciences, 113. 10.1029/2007jg000496

[gcb15740-bib-0082] Valentine, D. L. (2002). Biogeochemistry and microbial ecology of methane oxidation in anoxic environments: A review. Antonie van Leeuwenhoek, 81, 271–282. 10.1023/A:1020587206351 12448726

[gcb15740-bib-0083] Valentine, D. W. , Holland, E. A. , & Schimel, D. S. (1994). Ecosystem and physiological controls over methane production in Northern wetlands. Journal of Geophysical Research‐Atmospheres, 99(D1), 1563–1571. 10.1029/93jd00391

[gcb15740-bib-0084] Väliranta, M. , Salojarvi, N. , Vuorsalo, A. , Juutinen, S. , Korhola, A. , Luoto, M. , & Tuittila, E.‐S. (2017). Holocene fen‐bog transitions, current status in Finland and future perspectives. The Holocene, 27(5), 752–764. 10.1177/0959683616670471

[gcb15740-bib-0085] Walter, B. P. , & Heimann, M. (2000). A process‐based, climate‐sensitive model to derive methane emissions from natural wetlands: Application to five wetland sites, sensitivity to model parameters, and climate. Global Biogeochemical Cycles, 14(3), 745–765. 10.1029/1999gb001204

[gcb15740-bib-0086] Yavitt, J. B. , Lang, G. E. , & Downey, D. M. (1988). Potential methane production and methane oxidation rates in peatland ecosystems of the Appalachian Mountains, United States. Global Biogeochemical Cycles, 2(3), 253–268. 10.1029/GB002i003p00253

[gcb15740-bib-0087] Yavitt, J. B. , Yashiro, E. , Cadillo‐Quiroz, H. , & Zinder, S. H. (2012). Methanogen diversity and community composition in peatlands of the central to northern Appalachian Mountain region, North America. Biogeochemistry, 109(1–3), 117–131. 10.1007/s10533-011-9644-5

[gcb15740-bib-0088] Yrjälä, K. I. M. , Tuomivirta, T. , Juottonen, H. , Putkinen, A. , Lappi, K. , Tuittila, E.‐S. , Penttila, T. , Minkkinen, K. , Laine, J. , Peltoniemi, K. , & Fritze, H. (2011). CH_4_ production and oxidation processes in a boreal fen ecosystem after long‐term water table drawdown. Global Change Biology, 17(3), 1311–1320. 10.1111/j.1365-2486.2010.02290.x

[gcb15740-bib-0089] Zhang, H. , Piilo, S. R. , Amesbury, M. J. , Charman, D. J. , Gallego‐Sala, A. , & Valiranta, M. (2018). The role of climate change in regulating arctic permafrost peatland hydrological and vegetation change over the last millennium. Quaternary Science Reviews, 182, 121–130. 10.1016/j.quascirev.2018.01.003

[gcb15740-bib-0090] Zhang, H. , Tuittila, E.‐S. , Korrensalo, A. , Räsänen, A. , Virtanen, T. , Aurela, M. , Penttila, T. , Laurila, T. , Gerin, S. , Lindholm, V. , & Lohila, A. (2020). Water flow controls the spatial variability of methane emissions in a northern valley fen ecosystem. Biogeosciences, 17(23), 6247–6270. 10.5194/bg-17-6247-2020

[gcb15740-bib-0091] Zhang, H. , Väliranta, M. , Piilo, S. , Amesbury, M. J. , Aquino‐López, M. A. , Roland, T. P. , Salminen‐Paatero, S. , Paatero, J. , Lohila, A. , & Tuittila, E.‐S. (2020). Decreased carbon accumulation feedback driven by climate‐induced drying of two southern boreal bogs over recent centuries. Global Change Biology, 26(4), 2435–2448. 10.1111/gcb.15005 31961026

[gcb15740-bib-0092] Zhang, Z. , Zimmermann, N. E. , Stenke, A. , Li, X. , Hodson, E. L. , Zhu, G. F. , Huang, C. , & Poulter, B. (2017). Emerging role of wetland methane emissions in driving 21st century climate change. Proceedings of the National Academy of Sciences of the United States of America, 114(36), 9647–9652. 10.1073/pnas.1618765114 28827347 PMC5594636

