# Supplementary material for: Methane production and oxidation potentials along a fen‐bog gradient from southern boreal to subarctic peatlands in Finland
Source: Glob Chang Biol. 2021 Jun 28;27(18):4449–64. doi: 10.1111/gcb.15740 (PMC13420805; doi:10.1111/gcb.15740)
Supplement: Supplementary file 1 — Supplementary Material [file GCB-27--s001.pdf]

**Supplementary materials for:**

# Methane production and oxidation potentials along a fen-bog gradient from southern boreal to subarctic peatlands in Finland

**Hui Zhang[1,2*], Eeva-Stiina Tuittila[3], Aino Korrensalo[3], Anna M. Laine[3,4,5], Salli Uljas[3], Nina Welti[6,a], Johanna Kerttula[6], Marja Maljanen[6], David Elliott[7], Timo Vesala[1,8,9], Annalea Lohila[1,10]**

[1] Institute for Atmospheric and Earth System Research (INAR), Department of Physics, P.O. Box 68 (Pietari Kalmin katu 5), University of Helsinki, Helsinki, Finland

[2] Helsinki Institute of Sustainability Science (HELSUS), Helsinki, Finland

[3] Department of Forest Sciences, University of Eastern Finland, Joensuu, Finland

[4] Department of Ecology and Genetics, University of Oulu, Oulu, Finland

[5] Geological Survey of Finland, Kuopio, Finland

[6] Department of Environmental and Biological Sciences, University of Eastern Finland, Kuopio, Finland

[7] Environmental Sustainability Research Centre, University of Derby, Derby, UK

[8] Institute for Atmospheric and Earth System Research (INAR), Department of Forest Sciences, University of Helsinki, Helsinki, Finland

[9] Yugra State University, Khanty-Mansiysk, Russia

[10] Climate System Research, Finnish Meteorological Institute, Helsinki, Finland

[*] Correspondence to: Hui Zhang, huizhang_bj@163.com, ORCID: 0000-0002-3758-5722

[a] Current address: Commonwealth Scientific and Industrial Research Organization, Australia

**TEXT S1**

## Material and Methods

### *Methane flux data*

For all sites (with the exception of Halssiaapa), we compiled existing $CH_4$ flux data measured from a lawn habitat using manual chambers and averaged for the July-August period (Table S1). For Halssiaapa, the only available data was averaged from $CH_4$ fluxes that had been sampled in different habitats (flux data also included hummocks and hollows). The data represents the peak seasonal $CH_4$ emissions for the same habitat where samples were collected for laboratory incubation. The measurement/sampling period varied between sites; from one (e.g., Tervalamminsuo) to six summers (Lompolojänkkä).

**TABLE S1** July-August methane flux data for the studied peatlands. $T_5$: peat temperature at 5 cm depth.

| Site | Site code | Collecting year | Measured times | Mean $T_5$ (°C) | $CH_4$ flux data source |
|---|---|---|---|---|---|
| Tervalamminsuo | TE | 2020 | 4 | 19.9 | Unpublished data |
| Lakkasuo bog | LAB | 2000-2003 | 53 | 17.6 | Unpublished data |
| Siikaneva bog | SNB | 2012-2014 | 30 | 17.6 | Korrensalo et al., 2018 |
| Salmisuo | SA | 2007 | 58 | NA | Forbrich et al., 2011 |
| Siikajoki 6 | SJ6 | 2007 | 12 | 15.8 | Unpublished data |
| Siikaneva fen | SNF | 2004-2005 | 36 | 17.6 | Riutta et al., 2007 |
| Siikajoki 5 | SJ5 | 2007 | 12 | 16.0 | Unpublished data |
| Lakkasuo fen | LAF | 2000-2003 | 64 | 15.7 | Unpublished data |
| Siikajoki 3 | SJ3 | 2007 | 12 | 16.5 | Unpublished data |
| Halssiaapa | HA | 2014-2015 | 390 | 10.1* | Dinsmore et al., 2017 |
| Kittilä | KIT | 2013-2014 | 9 | 16.4 | Maljanen et al., 2018 |
| Lompolojänkkä | LO | 2005-2010 | 287 | 11.6 | Unpublished data |
| Kaamanen | KA | 2007 | 16 | 17.9 | Unpublished data |
| Kiposuo | KI | 2005-2006 | 24 | 14.1 | Juutinen et al., 2013 |

* Peat temperature was measured at a depth of 10 cm below the surface.

**TABLE S2** Peat properties and plant functional types (mean ± standard deviation) for three peatland types. Parameters that significantly differ between peatland types, based on mixed effect models, are shown in bold italic. Different letters indicate significant differences ($p < 0.05$).

| | Bog | Poor fen | Rich fen |
|---|---|---|---|
| Peat properties | | | |
| BD | 0.15 ± 0.03 | 0.15 ± 0.03 | 0.13 ± 0.02 |
| ***WHC*** | $7.00 \pm 1.88^{a}$ | $5.98 \pm 1.71^{ab}$ | $5.55 \pm 1.68^{b}$ |
| $\delta^{13}C$ | -26.70 ± 1.61 | -27.88 ± 0.83 | -28.52 ± 1.39 |
| ***LOI*** | $98.52 \pm 0.82^{a}$ | $95.38 \pm 0.66^{b}$ | $92.27 \pm 3.81^{c}$ |
| ***$Fe^{3+}$*** | $1.30 \pm 2.43^{a}$ | $9.82 \pm 13.73^{b}$ | $0.97 \pm 2.46^{a}$ |
| $Cl^-$ | 14.42 ± 8.24 | 32.12 ± 13.20 | 45.27 ± 65.78 |
| $NO_3^-$ | 2.04 ± 2.75 | 0.70 ± 1.71 | 2.82 ± 3.23 |
| $NH_4^+$ | 19.10 ± 13.77 | 5.57 ± 6.32 | 48.18 ± 77.70 |
| $PO_4^{3-}$ | 2.12 ± 3.32 | 2.00 ± 3.27 | 2.04 ± 4.34 |
| ***$SO_4^{2-}$*** | $10.95 \pm 6.30^{a}$ | $15.06 \pm 4.53^{ab}$ | $67.07 \pm 54.12^{b}$ |
| ***%N*** | $0.78 \pm 0.40^{a}$ | $1.16 \pm 0.29^{ab}$ | $1.51 \pm 0.63^{b}$ |
| ***C: N*** | $42.50 \pm 15.91^{a}$ | $18.05 \pm 5.45^{b}$ | $22.83 \pm 6.48^{b}$ |
| ***$\delta^{15}N$*** | $-3.22 \pm 1.15^{a}$ | $-2.08 \pm 0.46^{ab}$ | $-0.32 \pm 1.27^{b}$ |
| Plant functional types | | | |
| ***Sedge*** | $8.33 \pm 10.68^{a}$ | $11.00 \pm 9.14^{ab}$ | $17.90 \pm 12.53^{b}$ |
| ***Shrub*** | $5.17 \pm 2.88^{ab}$ | $8.00 \pm 5.51^{a}$ | $3.00 \pm 3.69^{b}$ |
| Herb | 2.53 ± 3.25 | 10.00 ± 12.51 | 8.57 ± 10.55 |
| ***Sphagnum*** | $92.21 \pm 15.35^{a}$ | $71.33 \pm 27.10^{a}$ | $23.52 \pm 30.66^{b}$ |
| ***Brown moss*** | $0.00 \pm 0.00^{a}$ | $0.00 \pm 0.00^{a}$ | $34.87 \pm 36.25^{b}$ |
| ***Aerenchyma*** | $10.87 \pm 10.56^{a}$ | $16.00 \pm 14.07^{ab}$ | $26.09 \pm 15.11^{b}$ |
| Vascular | 16.03 ± 9.22 | 29.00 ± 23.41 | 29.48 ± 15.14 |

*Notes*. BD is in g $cm^{-3}$, chemical compounds are in μg $g^{-1}$ dry soil, LOI and plant functional type cover are in %, and WHC is in g water $g^{-1}$ dry soil.

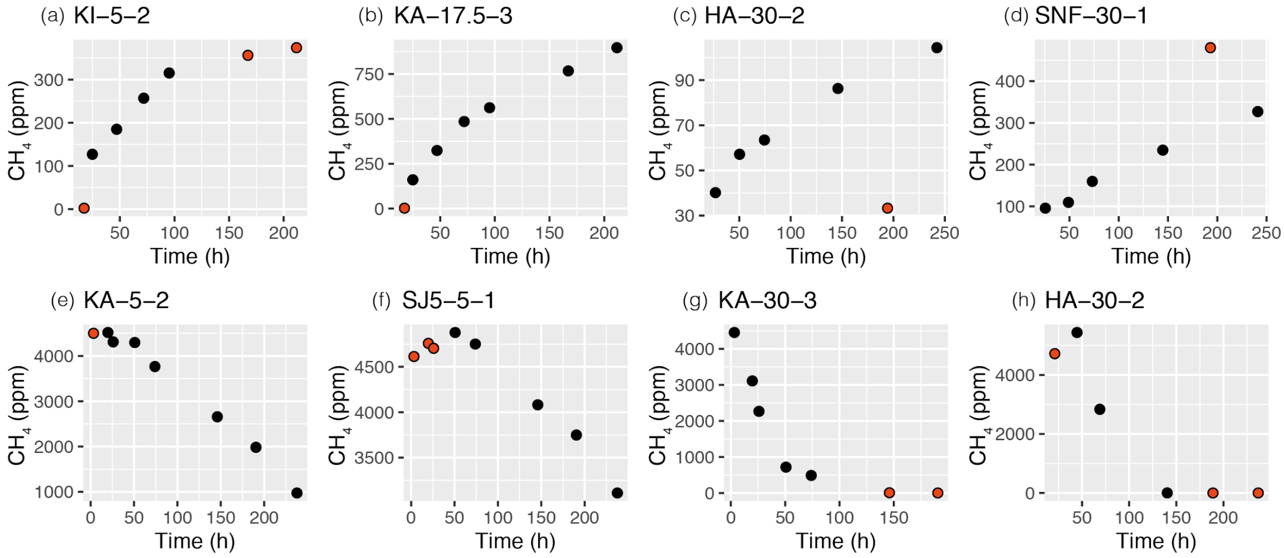


**FIGURE S1** Examples of the change in methane ($CH_4$) concentration over time for (a–d) $CH_4$ production and (e–f) oxidation measurements during the laboratory incubation process. Samples were labeled as site-treatment temperature-replication. Several patterns in $CH_4$ concentration changes were observed: (a) a linear increase following a short lag period, reaching asymptotic maximum after 100 hours, (b) a linear increase following a short lag period, (c, d) a linear increase with some failure in gas analysis, (e) a linear decrease following a short lag period, (f) a linear decrease with an initial increase, (g) a linear decrease, reaching zero between 100 and 150 hours, (h) a linear decrease with an initial increase, reaching zero between 125 and 150 hours. Calculations of $CH_4$ rates were based on the linear changes of the concentrations over time. Data points in red were excluded from the calculations.

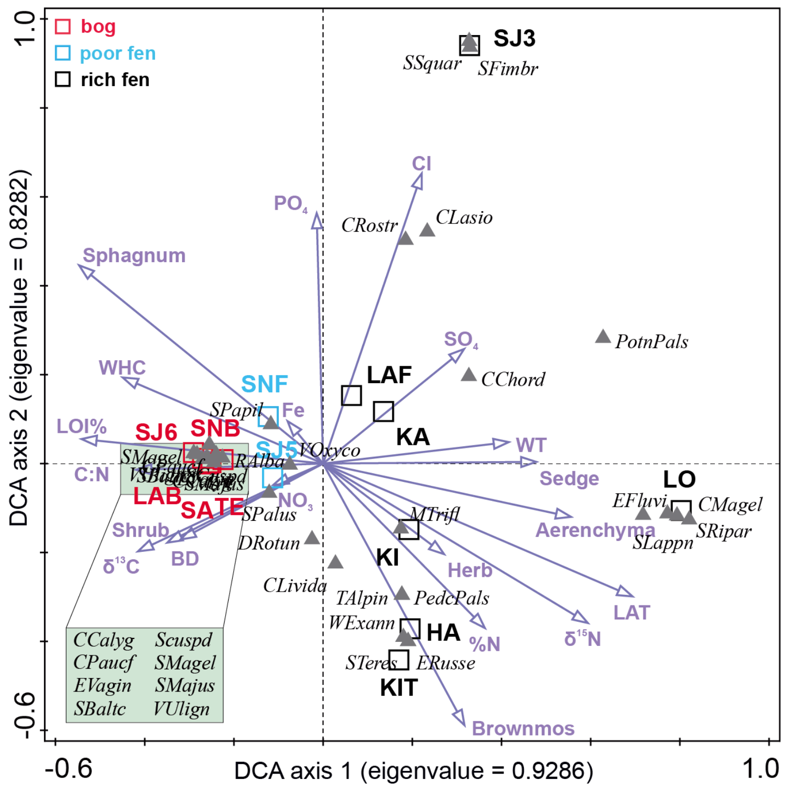


**FIGURE S2** Detrended correspondence analysis (DCA) based on plant community data with environmental data included as supplementary variables. The first two axes accounted for 22 % of the total variance in the vegetation. For clarity, the clustered plant species (green shading) are presented on the lower-left corner. Peatland types are indicated using red (bog), light blue (poor fen) and black (rich fen) site names. Full names of plant species and peat properties can be found in Table S3 and site codes are described in Table 1.

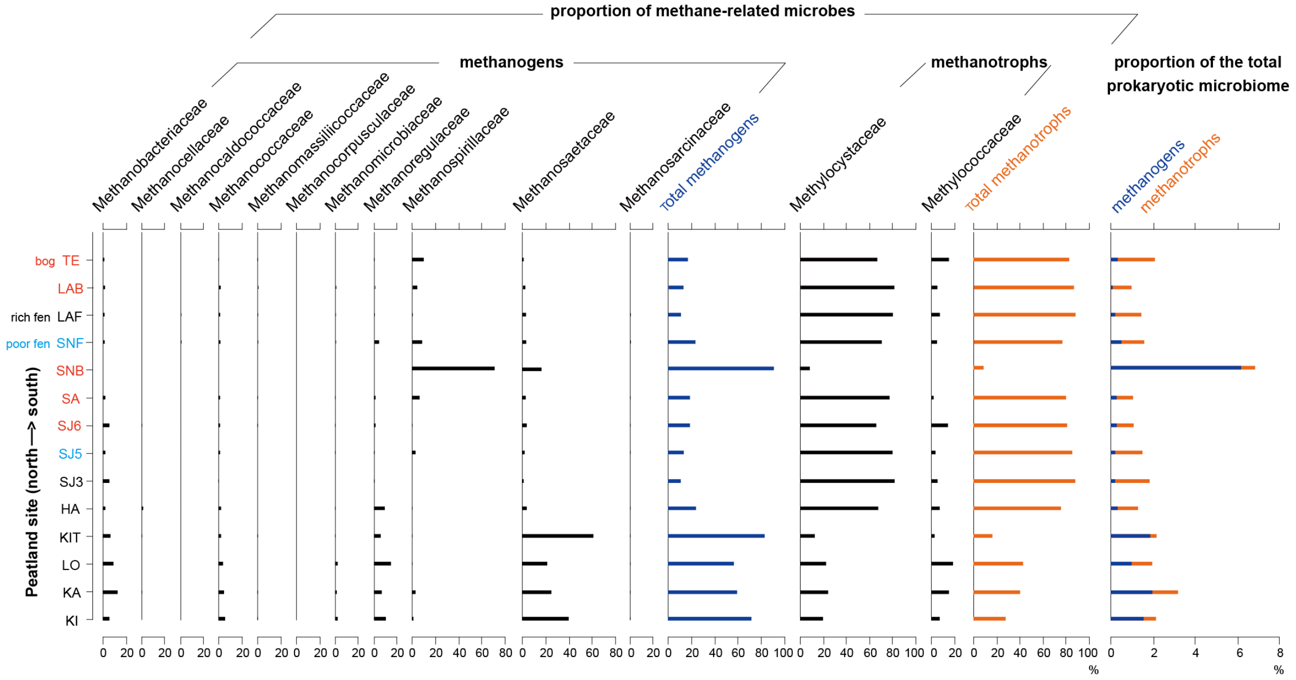


**FIGURE S3** The proportion of detected taxon-level DNA sequences of methane ($CH_4$)-related microbes, and the proportion of methanogens and methanotrophs of the total prokaryotic microbiome at each peatland site.

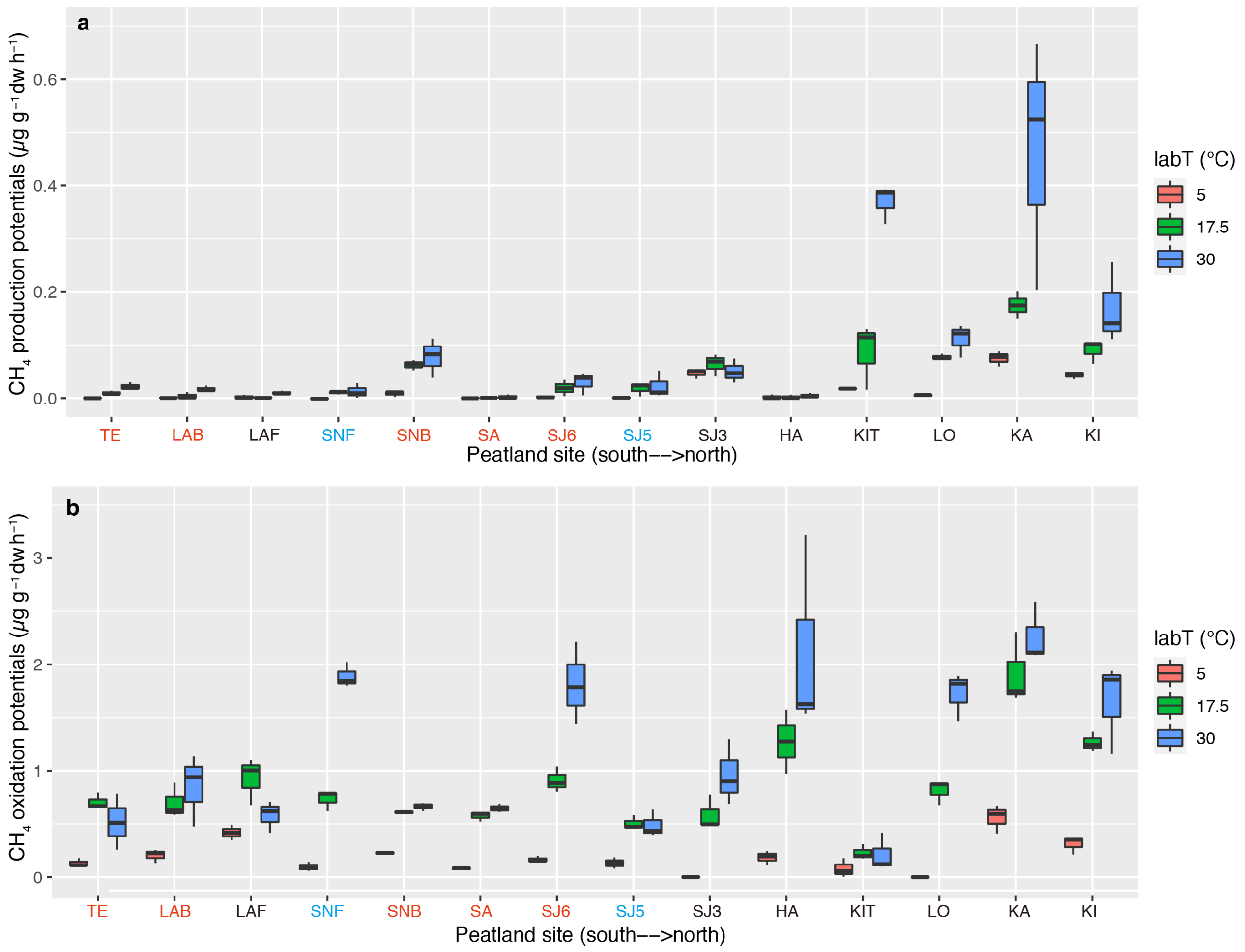


**FIGURE S4** Box plots showing (a) methane ($CH_4$) production and (b) oxidation potentials at laboratory temperatures (labT) of 5, 17.5 and 30 °C for each peatland site.

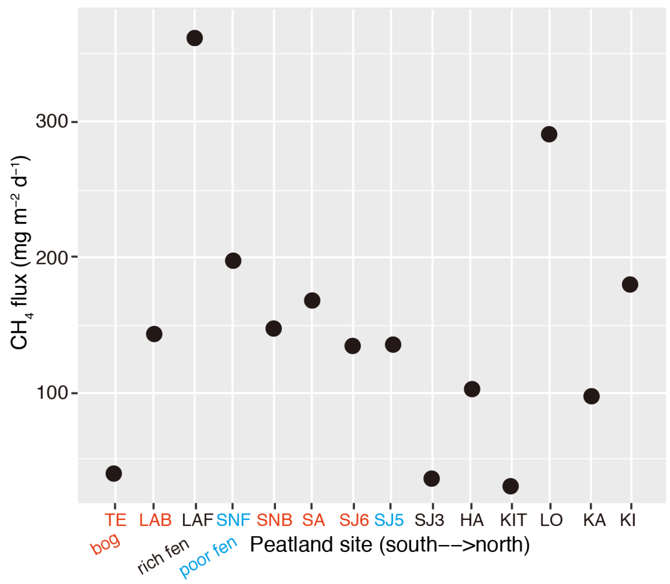


**FIGURE S5** Measured July-August methane ($CH_4$) fluxes for each peatland site. Note the flux data are compiled from previous studies, thus the measured points and years differ from the $CH_4$ production and oxidation potential measurements in this study.

**TABLE S3** Full names of plant taxa, microbial taxa, methane ($CH_4$) potential data and peat property variables shown in Figures 3, 4, 5, S1 and S2 and Table S2.

| Code | Full name | Code | Full name |
|---|---|---|---|
| CMagel | *Carex magellanica* | SPalus | *Stachys palustris* |
| CChord | *C. chordorrhiza* | TAlpin | *Thalictrum alpinum* |
| CLasio | *C. lasiocarpa* | VOxyco | *Vaccinium oxycoccos* |
| CLivida | *C. livida* | VUlign | *V. uliginosum* |
| CPaucf | *C. pauciflora* | CCalyg | *Chamaedaphne calygulata* |
| CRostr | *C. rostrata* | SBaltc | *Sphagnum balticum* |
| DRotun | *Drosera rotundifolia* | SCuspd | *S. cuspidatum* |
| EFluvi | *Equisetum fluviatile* | SFimbr | *S. fimbriatum* |
| ERusse | *Eriophorum russeolum* | SMagel | *S. magellanicum* |
| EVagin | *E. vaginatum* | SMajus | *S. majus* |
| MTrifl | *Menyanthes trifoliata* | SPapil | *S. papillosum* |
| PedcPals | *Pedicularis palustris* | SRipar | *S. riparium* |
| PotnPals | *Potentilla palustris* | SSquar | *S. squarrosum* |
| RAlba | *Rhynchospora alba* | STeres | *S. teres* |
| SLappn | *Salix lapponum* | WExann | *Warnstorfia exannulata* |
| $CH_4$_O_5 | $CH_4$ oxidation at 5 °C | $CH_4$_A_5 | $CH_4$ production at 5 °C |
| $CH_4$_O_17 | $CH_4$ oxidation at 17.5 °C | $CH_4$_A_17 | $CH_4$ production at 17.5 °C |
| $CH_4$_O_30 | $CH_4$ oxidation at 30 °C | $CH_4$_A_30 | $CH_4$ production at 30 °C |
| BD | Bulk density (g $cm^{-3}$) | $PO_4$ | µg $PO_4^{3-}$ $g^{-1}$ dw |
| C:N | Carbon/nitrogen mass ratio | $SO_4$ | µg $SO_4^{2-}$ $g^{-1}$ dw |
| %N | Nitrogen content | Cl | µg $Cl^-$ $g^{-1}$ dw |
| LOI | Loss on ignition (%) | Fe | µg $Fe^{3+}$ $g^{-1}$ dw |
| $\delta^{13}C$ | Isotope $^{13}C/^{12}C$ ratio (‰) | WHC | Water holding capacity (g water $g^{-1}$ dw) |
| $\delta^{15}N$ | Isotope $^{15}N/^{14}N$ ratio (‰) | WT | Water table (cm) |
| $NO_3$ | µg $NO_3^-$ $g^{-1}$ dry weight soil (dw) | LAT | Latitude |
| NH4 | µg $NH_4^+$ $g^{-1}$ dw | MetOcc | Methylococcaceae |
| MetMic | methanomicrobiaceae | MetYst | Methylocystaceae |
| MetCor | methanocorpusculaceae | MetBac | Methanobacteriaceae |
| MetReg | methanoregulaceae | MetCoc | methanoceccaceae |
| MetCal | methanocaldococcaceae | MetSae | methanosaetaceae |
| MetSar | methaneosarcinaceae | MetCel | methanocellaceae |
| MetMas | methanomassiliicoccaceae | MetSpi | methanospirillaceae |

**TABLE S4** Parameter estimates of the linear mixed-effect models of (a) impact of peatland type on temperature response of methane ($CH_4$) production and oxidation, and (b) impact of peat property, plant functional type and microbial community on temperature response of $CH_4$ production and oxidation.

| (a) | Coeff. | SE | DF | *t*-value | *P*-value |
|---|---|---|---|---|---|
| $CH_4$ production (AIC = -501) | | | | | |
| **Fixed part** | | | | | |
| ***Bog as reference*** | | | | | |
| Intercept | -0.00085 | 0.01190 | 81 | -0.07142 | 0.9432 |
| labT | 0.00047 | 0.00031 | 81 | 1.48004 | 0.1427 |
| Poor fen | 0.00037 | 0.02226 | 11 | 0.01652 | 0.9871 |
| Rich fen | -0.00043 | 0.01558 | 11 | -0.02744 | 0.9786 |
| labT: Poor fen | -0.00016 | 0.00059 | 81 | -0.27730 | 0.7823 |
| labT: Rich fen | 0.00157 | 0.00041 | 81 | 3.81763 | 0.0003 |
| ***Poor fen as reference*** | | | | | |
| Intercept | -0.00048 | 0.01882 | 81 | -0.02563 | 0.9796 |
| labT | 0.00030 | 0.00050 | 81 | 0.60795 | 0.5449 |
| Bog | -0.00037 | 0.02227 | 11 | -0.01652 | 0.9871 |
| Rich fen | -0.00080 | 0.02134 | 11 | -0.03728 | 0.9709 |
| labT: Bog | 0.00016 | 0.00059 | 81 | 0.27730 | 0.7823 |
| labT: Rich fen | 0.00174 | 0.00056 | 81 | 3.07737 | 0.0028 |
| **Random part** | | | | | |
| SD (site) | 0.02248 | | | | |
| SD (sample in site) | <0.00001 | | | | |
| Residual SD | 0.02154 | | | | |
| $CH_4$ oxidation (AIC = -194) | | | | | |
| **Fixed part** | | | | | |
| ***Bog as reference*** | | | | | |
| Intercept | 0.04609 | 0.04316 | 81 | 1.06792 | 0.2887 |
| labT | 0.00800 | 0.00113 | 81 | 7.08802 | 0.0000 |
| Poor fen | -0.04461 | 0.08074 | 11 | -0.55247 | 0.5917 |
| Rich fen | -0.00623 | 0.05651 | 11 | 0.11026 | 0.9142 |
| labT: Poor fen | 0.00278 | 0.00211 | 81 | 1.31891 | 0.1909 |
| labT: Rich fen | 0.00258 | 0.00148 | 81 | 1.74455 | 0.0849 |
| ***Poor fen as reference*** | | | | | |
| Intercept | 0.00148 | 0.06824 | 81 | 0.02172 | 0.9827 |
| labT | 0.01078 | 0.00178 | 81 | 6.04341 | 0.0000 |
| Bog | 0.04461 | 0.08074 | 11 | 0.55247 | 0.5917 |
| Rich fen | 0.05084 | 0.0.07738 | 11 | 0.65702 | 0.5247 |
| labT: Bog | -0.00278 | 0.00211 | 81 | -1.31891 | 0.1909 |
| labT: Rich fen | -0.00021 | 0.00202 | 81 | -0.10224 | 0.9188 |
| **Random part** | | | | | |
| SD (site) | 0.08186 | | | | |
| SD (sample in site) | <0.00001 | | | | |
| Residual SD | 0.07725 | | | | |

| (b) | Coeff. | SE | DF | *t*-value | *P*-value |
|---|---|---|---|---|---|
| $CH_4$ production | | | | | |
| **Fixed part** | | | | | |
| Intercept | 0.0004 | 0.0108 | 81 | -0.033 | 0.9734 |
| labT | 0.0005 | 0.0003 | 81 | 1.370 | 0.1744 |
| PCA 1 axis score | -0.0046 | 0.0067 | 27 | -0.693 | 0.4940 |
| methanogens | -0.0037 | 0.0256 | 12 | -0.145 | 0.8868 |
| labT: PCA 1 axis score | 0.00091 | 0.0002 | 81 | 4.014 | 0.0001 |

| | | | | | |
|---|---|---|---|---|---|
| labT: methanogens | 0.0021 | 0.0008 | 81 | 2.595 | 0.0112 |
| **Random part** | | | | | |
| SD (site) | 0.0159 | | | | |
| SD (sample in site) | <0.0001 | | | | |
| Residual SD | 0.0178 | | | | |
| **Marginal ANOVA test of the model** | | | | | |
| | DF | F-value | *p*-value | | |
| Intercept | 81 | 0.0011 | 0.9734 | | |
| labT | 81 | 1.8772 | 0.1744 | | |
| PCA 1 axis score | 27 | 0.4808 | 0.4940 | | |
| methanogens | 12 | 0.0212 | 0.8868 | | |
| labT: PCA 1 axis score | 81 | 16.1105 | 0.0001 | | |
| labT:methanogens | 81 | 6.7343 | 0.0112 | | |
| $CH_4$ oxidation | | | | | |
| **Fixed part** | | | | | |
| Intercept | -0.1777 | 0.0852 | 79 | -2.086 | 0.0402 |
| labT | 0.0338 | 0.0038 | 79 | 8.790 | <0.0001 |
| $labT^2$ | -0.0004 | 0.0001 | 79 | -5.301 | <0.0001 |
| WHC | 0.0139 | 0.0094 | 24 | 1.487 | 0.1500 |
| %N | 0.0309 | 0.0365 | 24 | 0.846 | 0.4057 |
| Aerenchyma | -0.0008 | 0.0009 | 24 | -0.841 | 0.4083 |
| SO4 | 0.0008 | 0.0004 | 24 | 2.057 | 0.0507 |
| labT: WHC | -0.0012 | 0.0003 | 79 | -3.714 | 0.0004 |
| labT: %N | -0.0038 | 0.0009 | 79 | -3.908 | 0.0002 |
| labT: Aerenchyma | 0.0001 | <0.001 | 79 | 2.614 | 0.0107 |
| **Random part** | | | | | |
| SD (site) | 0.0700 | | | | |
| SD (sample in site) | <0.0001 | | | | |
| Residual SD | 0.0630 | | | | |
| **Marginal ANOVA test of the mode** | | | | | |
| | DF | F-value | *p*-value | | |
| Intercept | 79 | 4.3523 | 0.0402 | | |
| Aerenchyma | 24 | 0.7084 | 0.4083 | | |
| labT | 79 | 77.2721 | <0.0001 | | |
| $labT^2$ | 79 | 28.0984 | <0.0001 | | |
| %N | 24 | 0.7164 | 0.4057 | | |
| SO4 | 24 | 4.2312 | 0.0507 | | |
| WHC | 24 | 2.2115 | 0.1500 | | |
| labT:Aerenchyma | 79 | 6.8231 | 0.0107 | | |
| labT:%N | 79 | 15.2739 | 0.0002 | | |
| labT:WHC | 79 | 13.7974 | 0.0004 | | |

## References


Korrensalo, A., Männistö, E., Alekseychik, P., Mammarella, I., Rinne, J., Vesala, T., & Tuittila, E.-S. (2018). Small spatial variability in methane emission measured from a wet patterned boreal bog. *Biogeosciences, 15*(6), 1749-1761. https://doi.org/10.5194/bg-15-1749-2018

Forbrich, I., Kutzbach, L., Wille, C., Becker, T., Wu, J., & Wilmking, M. (2011). Cross-evaluation of measurements of peatland methane emissions on microform and ecosystem scales using high-resolution land cover classification and source weight modelling. *Agricultural and Forest Meteorology, 151*(7), 864-874. https://doi.org/10.1016/j.agrformet.2011.02.006

Dinsmore, K.J., Drewer, J., Levy, P.E., George, C., Lohila, A., Aurela, M., & Skiba, U.M. (2017). Growing season $CH_4$ and $N_2O$ fluxes from a subarctic landscape in northern Finland; from chamber to landscape scale. *Biogeosciences*, *14*, 799-815. https://doi.org/10.5194/bg-14-799-2017

Maljanen, M., Kujala, K., Reinikainen, J., Tuittila, E.-S., & Ronkanen, A.-K. (2018). Greenhouse gas dynamics of a northern boreal peatland used for treating metal mine wastewater. *Wetlands*, *38*, 905-917. https://doi.org/10.1007/s13157-018-1040-7

Juutinen, S., Väliranta, M., Kuutti, V., Laine, A. M., Virtanen, T., Seppä, H., Weckstrom, J., & Tuittila, E.-S. (2013). Short-term and long-term carbon dynamics in a northern peatland-stream-lake continuum: A catchment approach. *Journal of Geophysical Research: Biogeosciences, 118*(1), 171-183. https://doi.org/10.1002/ jgrg.20028

Riutta, T., Laine, J., Aurela, M., Rinne, J., Vesala, T., Laurila, T., Haapanala, S., Pihlatie, M., & Tuittila, E.-S. (2007). Spatial variation in plant community functions regulates carbon gas dynamics in a boreal fen ecosystem. *Tellus Series B-Chemical and Physical Meteorology, 59*(5), 838-852. https://doi.org/10.1111/j.1600-0889.2007.00302.x
